# Immersive Virtual Reality–Assisted Therapy for Distressing Voices in Psychosis: Qualitative Study of Participants’ and Therapists’ Experiences in the Challenge Trial

**DOI:** 10.2196/77920

**Published:** 2025-12-01

**Authors:** Mads Juul Christensen, Matilde Poulsen Rydborg, Rikke Jørgensen, Cecilie Dueholm Nielsen, Jan Mainz, Imogen H Bell, Neil Thomas, Lisa Charlotte Smith, Lise Sandvig Mariegaard, Thomas Ward, Merete Nordentoft, Louise Birkedal Glenthøj, Ditte Lammers Vernal

**Affiliations:** 1 Psychiatry, Aalborg University Hospital, North Denmark Region, Brandevej 5, Aalborg, 9220, Denmark, 0045 20551002; 2 Department of Clinical Medicine, Aalborg University, Aalborg, Denmark; 3 Unit for Psychiatric Research, Psychiatry, Aalborg University Hospital, North Denmark Region, Aalborg, Denmark; 4 Department of Health Economics, University of Southern Denmark, Odense, Denmark; 5 Orygen, The National Centre of Excellence in Youth Mental Health, Parkville, Australia; 6 Centre for Youth Mental Health, The University of Melbourne, Parkville, Australia; 7 Centre for Mental Health and Brain Sciences, Swinburne University of Technology, Melbourne, Australia; 8 Alfred Hospital, Melbourne, Australia; 9 VIRTU Research Group, Mental Health Services CPH, Copenhagen University Hospital – Mental Health Services CPH, Copenhagen, Denmark; 10 Department of Clinical Medicine, Faculty of Health and Medical Sciences, University of Copenhagen, Copenhagen, Denmark; 11 Department of Psychology, Institute of Psychiatry, Psychology & Neuroscience, King's College London, London, United Kingdom; 12 South London and Maudsley NHS Foundation Trust, London, United Kingdom; 13 Research Centre for Mental Health (CORE), Mental Health Centre Copenhagen, Copenhagen University Hospital – Mental Health Services CPH, Copenhagen, Denmark; 14 Department of Psychology, University of Copenhagen, Copenhagen, Denmark

**Keywords:** virtual reality, virtual reality–assisted therapy, AVATAR therapy, voice-hearing, auditory verbal hallucinations, psychosis, schizophrenia, implementation outcomes, acceptability, feasibility, semi-structured interviews

## Abstract

**Background:**

Immersive virtual reality–assisted therapy (VRT) is a relational therapy for distressing voices in psychosis. Like AVATAR therapy (AT), VRT centers on therapist-facilitated dialogues with a digital avatar representing a voice. Unlike AT, VRT uses immersive virtual reality (VR). While participant experiences of AT have been explored, therapist perspectives remain unexamined, and for VRT, neither participant nor therapist experiences have been studied. Understanding these perspectives is essential to inform optimization of therapy, future research, and implementation.

**Objective:**

The objective of this qualitative study was to explore both trial participants’ and therapists’ experience of VRT in the Challenge trial.

**Methods:**

Semistructured interviews were conducted with 10 trial participants and 8 therapists across the 3 Challenge trial sites. Trial participants were purposively sampled to ensure site representation and variation in voice-hearing duration. Individual interviews were conducted with trial participants, while therapists participated in site-based groups with 2‐3 in each. Interviews were audio-recorded, transcribed, and subjected to reflexive thematic analysis from a critical realist position. Coding and theme development were inductive. People with lived experience were invited to an initial focus group for topic guide development and a later theme validation workshop. Reporting followed the Standards of Reporting Qualitative Research (SRQR).

**Results:**

A total of five overarching themes were generated: (1) using technology to meet the voice, (2) a different approach to voice-hearing and treatment, (3) on a tight schedule, (4) a toolbox for transformation, and (5) a price to pay. Trial participants and therapists generally found VRT acceptable, appropriate, and feasible. Highlights included the acknowledging approach to the voice(s), facilitation of engagement with the voice(s), and opportunity to share the otherwise private experience of voice-hearing. Externalizing and embodying the voice(s) in VR-supported avatar role-plays was seen as a key affordance. Positive outcomes included increased trial participant empowerment and self-worth, enabling or improving voice dialogue, new understanding of voice intentions, and changes in voice frequency or content. Challenges included instances of participant anxiety, exhaustion, or suboptimal sense of (voice) presence; adverse voice reactions; technological malfunctions and limitations to avatar design; measurement insensitivity; tensions between assertiveness and compassion; difficulties with reproducing negative voice content; and the demanding nature of the therapy and the nontraditional skills required of therapists.

**Conclusions:**

The study provided comprehensive insights into trial participants’ and therapists’ experiences of VRT in the Challenge trial. Findings share several similarities with qualitative research on other relational therapies for distressing voices and highlight VRT’s potential for positive change. Key considerations for future research and implementation include monitoring anxiety and voice reactions, ensuring operational reliability of hardware and software, and addressing the additional effort required by therapists, which may be unsustainable in routine practice. As a demanding intervention, the successful implementation of VRT will require adequate training, supervision, and structural support.

## Introduction

Hearing voices (auditory verbal hallucinations) is a phenomenon occurring in the general population, across various disorders, and commonly associated with psychosis or schizophrenia spectrum disorder diagnoses [1-4]. Voices may be highly distressing [5], especially due to appraisals of the meaning and intent of voices [6] and negative content [7,8].

Within psychosis, an estimated 1 in 3 individuals have a suboptimal pharmacological treatment response [9,10] with side effects being common [11]. Cognitive behavioral therapy for psychosis (CBTp) is recommended as a first-line psychological treatment in several national guidelines [e.g., 12] and shows a small-to-moderate effect on hallucinations [13]. However, CBTp has traditionally focused on psychosis more broadly rather than hearing voices specifically, limiting its sensitivity [14].

More targeted approaches have evolved that focus on voices more specifically [15-17]. Relational therapies are a promising approach for hearing voices, aiming to modify the interaction patterns and dynamics between the hearer and the voice through experiential dialogue with voice-related identities [18]. These include Avatar Therapy (AT) [19,20], Relating Therapy (RT) [21-25], and Talking with Voices (TwV) [26-28]. AT engages an individual in computer-assisted on-screen dialogues with a digital avatar representing the most distressing voice. In the AVATAR trial, therapists alternated between real-time voice transformation to role-play the avatar and speaking in their own voice, targeting therapeutic goals such as power, control, experiential disengagement, and compassion [29]. The AVATAR2 trial demonstrated that both brief (Avatar brief version, AV-BRF) and extended (Avatar extended version, AV-EXT) AT effectively reduced voice severity and distress [30]. AV-BRF focused on exposure, assertiveness, and self-esteem. AV-EXT emphasized understanding voices within the context of the individual’s life history [31] and allowed greater scope to address broader treatment targets, including trauma [30,32]. In an early value assessment, the use of AT has been recommended to collect real-world evidence and cost-effectiveness [33].

Virtual reality (VR) offers a promising avenue for improving treatment outcomes in mental health [34] and psychosis [35]. In VR, the central concept of immersion refers to the extent to which users feel mentally and emotionally absorbed within virtual environments, and key influencing factors include an immersive system, a sense of presence, and ecological validity [34]. The immersive system—the technical aspects of the VR equipment and software—can influence sense of presence (henceforth presence) [36]—the subjective feeling of “being there” in a virtual environment [37]. Despite terminological ambiguity [37],presence may enhance therapeutic responses [38,39], when delivered optimally [40]. In nonimmersive AT, the concept of presence has been adapted to “sense of voice presence” (henceforth voice presence) to more accurately capture the subjective experience of dialoguing with the voice [41]. Voice presence was consistently observed during AVATAR sessions, with an interaction between reduced anxiety and strong presence being implicated in outcomes [41]. As presence is stronger in VR than nonimmersive formats [34], adding VR to AT may optimize outcomes. Thus, a variant of AT using VR and a separate software called virtual reality–assisted therapy (VRT) for distressing voices has been developed and shown promising results [42]. The recent Challenge trial demonstrated VRT’s efficacy in reducing voice severity posttreatment [43], with impact of avatar features and “sense of spatial presence” (henceforth spatial presence; how real it felt to be in the virtual room with the avatar) on treatment outcomes reported elsewhere [44]. A modified VRT version has been explored in a Challenge substudy (K Rasmussen, unpublished data, 2025).

VRT avatar dialogues can evoke a range of emotional responses [45], with findings suggesting that negative emotions decrease while positive emotions increase as sessions progress [46]. These emotional shifts may be driven by changes in relational dynamics, as reflected in evolving discourse patterns [47]. Anxiety levels are initially high [46,48], with early sessions often perceived as stressful [48]. The initial phase is confrontational, and withdrawal and dropout rates are higher initially than in later phases [29,49]. Although adverse emotional responses are expected, too intense anxiety or distress may reduce tolerability. Indeed, for 29% of the Challenge trial participants, adjustments were made to the pace or duration of exposures in response to reports of anxiety [43]. Assessing unwanted events—such as therapy prolongation or symptom exacerbation—is essential for ensuring that therapies remain safe [50-52].

Digital mental health interventions risk a research-to-practice gap [53,54], making evaluation of implementation outcomes crucial for translation into routine care [55,56]. AT studies have reported acceptability [29,57] and feasibility [30,58], and a VRT study has addressed both [48]. However, uptake in routine care remains uncertain, as clinical and implementation research differ [59]. While dedicated implementation studies are needed, qualitative research within clinical trials can prove an initial window into lived experience and stakeholder perspectives [60], informing future implementation efforts [61-63]. Incorporating service-user perspectives is particularly valuable for optimizing therapy [64-66]. Implementation science outcomes, particularly acceptability, appropriateness, and feasibility [56], may provide a useful basis for interpreting experiences of VRT and identifying key considerations for implementation.

Participants’ experiences of AT have been explored qualitatively in AVATAR1 and AVATAR2. The former found AT acceptable and beneficial, though some participants reported strong emotional reactions and trauma-related memories that did not disrupt therapy [57]. The latter, currently being prepared for publication, examined direct early work with verbatim voice content and the role of the therapeutic alliance [67]. In contrast, no equivalent studies have explored the experiences of VRT participants.

Therapists’ experiences have not yet been explored in either AT or VRT. Considering dissemination and implementation, it is crucial to account for the perspectives of clinicians delivering these therapies [68]. This is especially important for VR-based interventions, where mental health professionals have raised concerns regarding technological limitations, potential side effects, and increased time and workload demands [69]. Furthermore, as demonstrated in both AT [20,29] and VRT [49], conducting avatar dialogues requires a nontraditional therapeutic skill set, particularly the “actor-like” components that pose unique challenges.

This study aimed to explore the experiences of trial participants and therapists with VRT in the Challenge trial and to interpret the findings through the lens of implementation outcomes.

## Methods

### Design and Setting

This qualitative study was part of Challenge, a Danish multicenter randomized controlled trial (RCT) on VRT for distressing voices (November 2020–June 2024). The VRT intervention comprised 7 sessions over 12 weeks (1 design session and 6 avatar dialogue sessions), inspired by the original AT protocol [29,70], with 2 booster sessions between weeks 12 and 24. Avatar dialogues were delivered using a head-mounted display (HMD), with participant and therapist present in the same location. A panic button allowed participants to remove the avatar. Elsewhere, more details are reported on Challenge’s methodology [70] and outcomes [43]. Secondary analyses of trial participants’ childhood trauma configurations and their relationships with voice-related distress are reported in a separate study [71].

Methodologically oriented toward pragmatism [72-74], this study adopted a practical and flexible approach to explore participants’ and therapists’ experiences. Semistructured interviews [75] were conducted at the 3 trial sites: a research unit in Copenhagen and 2 outpatient clinics in Aalborg and Esbjerg offering Early Intervention Services (OPUS) [76] or Flexible-Assertive Community Treatment (F-ACT) [77]. Reporting adheres to the standards for reporting qualitative research (SPQR) [78].

### Trial Participant Selection

In this study, both voice-hearing individuals (trial participants) and therapists are considered participants. To differentiate, “trial participants” refers specifically to those receiving VRT. A multistage, purposeful sampling strategy [79] was used to ensure variation in voice-hearing duration and site representation, with interviews conducted within a year of therapy completion (mean 7.55 months from baseline). Participants were contacted by phone and therapists by email.

Sample size was guided by the concept of information power [80] and balanced with practical constraints. Information power was appraised throughout the interview process, leading to the conclusion of recruitment after interviewing 10 trial participants and 8 therapists. At this point, authors MJC and MPR judged the sample to hold sufficient information to elucidate the study’s aims. There is no minimum sample size for thematic analysis [81]. However, a model of data saturation has recently been proposed [82], published only after the current study’s interview phase. Although saturation was not a guiding principle due to debates about its compatibility with reflexive thematic analysis [83], the study’s sample falls within the range estimated to be sufficient for achieving saturation [84].

Of the 21 trial participants invited, 10 were included, while the remaining were unavailable (n=5), unreachable (n=3), excluded due to acute psychosis (n=1), or declined without explanation (n=3). All trial participants met the Challenge trial’s inclusion criteria, including ages 18 years and older, a schizophrenia spectrum disorder diagnosis (excluding schizophrenia simplex and schizotypal disorder; *International Classification of Diseases-10*), persistent auditory hallucinations (≥3 months, Scale for the Assessment of Positive Symptoms [SAPS] score ≥3), in Danish psychiatric care, able to consent, stable antipsychotic regimen ≥4 weeks, insufficient response to current or ≥2 past antipsychotics if no current antipsychotic regimen [70]. Of the 11 eligible therapists, 8 still active in the trial agreed to participate; 3 were no longer involved or unreachable.

### Individual Interview and Data Collection

Trial participants were interviewed individually, while therapists were interviewed in site-based groups, with 2‐3 in each. Interview guides were developed in mid-2022 based on (1) a lived experience focus group with trial participants (n=4) not involved in the current qualitative interviews and (2) two focus groups with therapists (n=6) from two trial sites. After feedback from research members (3 of 6 responded) and external VR experts (1 of 3 responded), a pilot trial participant interview was conducted and later included among the final 10. While the guides were continuously revised, only minor changes were made to improve clarity, ensuring consistency across interviews (see MultimediaAppendices 1  2 for English versions).

Trial participants could bring a peer or case manager, resulting in one interview being with the presence of nonparticipants. Interviews were audio-recorded and transcribed with GoodTape AI [85]. Author MPR proofread, anonymized, and verified the transcripts for accuracy. Trial participant interviews (March 2023-October 2023) lasted 51:34-112:46 minutes (mean 86:03 min), and therapist group interviews (April 2023-February 2024) lasted 140:21–148:06 minutes (mean 145:11 min).

Background information on trial participants was obtained from the main trial records and supplemented during the interview, while therapist information was collected during the interviews and through follow-up email correspondence.

### Research Team and Reflexivity

The research team had clinical and, in most cases, research experience with psychotic disorders, with backgrounds in nursing, clinical psychology, or medicine. Most were involved in the Challenge trial or employed at participating mental health organizations. Data collection was conducted by team members experienced in clinical interviews, though with varying qualitative research experience. The first author led the analysis, supervised by author RJ, a qualitative research specialist. Reflexivity was an ongoing process, addressing how previous relationships, roles, and disciplinary backgrounds might shape interpretation. RJ, who was not involved in the trial, provided independent oversight to strengthen the reflexive approach and to prevent trial-involved team members from making interpretations not fully supported by the data. A total of 2 authors who were also therapist interviewees did not participate in analysis. Some interviewers were professionally acquainted with participants. Notable dependent relationships included: (1) the first author having conducted a research assessment of a trial participant as part of the Challenge RCT; (2) the last author being both a therapist interviewee and manager of the authors MJC, MPR, and CDN; and (3) interviewers being colleagues—near or distant—of interviewed therapists. These relationships and associated hierarchical dynamics were openly discussed regarding their impact on data collection and analysis. Reflexivity was supported through notes, memos, collaborative coding, and critical discussions of diverging interpretations, enabling a reflective and iterative analytic process.

### Data Analysis

Data were analyzed thematically, informed by reflexive thematic analysis [86-90], as it emphasizes researcher reflexivity and treats subjectivity as a resource. A critical realist position was adopted to locate and make sense of trial participants’ and therapists’ descriptions of their experiences of VRT. Critical realism theorizes an independent truth as possible but unreachable due to each individual’s different locatedness and perspectives [91]. Coding and theme development were inductive to remain grounded in participants’ accounts, while recognizing that it was inevitably shaped by underlying theoretical assumptions [92], including (but not limited to) the research team’s optimism about VRT’s efficacy [43] and intention to interpret themes in relation to implementation outcomes [56]. The 6-stage analysis progressed from data familiarization, open coding, and initial theme identification (stages 1‐3) to theme refinement and presentation (stages 4‐6). In stages 1‐2, authors MJC, MPR, and CDN familiarized themselves with the data and coded transcripts, with participant interviews coded by authors MJC and CDN, and therapist interviews by MJC and MPR. Stage 3 involved developing initial themes separately for trial participant (authors MJC and CDN) and therapist (authors MJC and MPR) transcripts. Stage 4 involved the reinvestigation and refinement of initial separate themes. Stages 3‐4 were repeated across the entire material, integrating trial participant and therapist materials (authors MJC and MPR). Information power was deemed sufficient as no new insights were generated in the final trial participant interview, and all available therapists had been interviewed. Coding was managed using Nvivo (version 14; Lumivero), and codes were collapsed into themes using online whiteboard Miro. Stage 5 involved clearly defining and naming the final themes. The processes in stages 3‐5 involved ongoing discussions among authors MJC, MPR, and CDN, supervised by RJ. Stage 6 involved writing up the analysis, with participant quotes used for illustration (see the Thematic Analysis subsection in the Results section ). For theme validation, all Challenge experiment group participants from one trial site (n=44) were invited to a workshop to discuss the findings (see Multimedia Appendix 3).

### Ethical Considerations

The Challenge RCT was approved by the Committee on Health Research Ethics of the Capital Region of Denmark and the Danish Data Protection Agency (Project ID: H-19086621). This study adhered to the Declaration of Helsinki, with no additional approval required. All participants received written information and provided written informed consent. Confidentiality was maintained; however, participants were informed that any disclosure of significant risks during interviews would be shared with their care teams. All interview data were anonymized before analysis to protect participant privacy. No compensation was provided.

## Results

### Demographics

The sample included 10 trial participants (Participants 1-10) who had completed six (n=1) or all seven (n=9) VRT sessions, and eight therapists (Therapists 1-8) from the Challenge trial across sites (see Table 1).

**Table 1.** Background information of interviewed Challenge-virtual reality–assisted therapy (VRT) trial participants (demographic, clinical, and trial-related characteristics) and therapists (demographic and professional characteristics).

Characteristics	n (%)	Mean (SD)
Participants		
Age (years)	—^a^	34.5 (13.6)
Gender		
Men	3 (30)	—
Women	6 (60)	—
Other	1 (10)	—
Ethnicity		
White	10 (100)	—
Black or mixed Black	0 (0)	—
South Asian or mixed South Asian	0 (0)	—
Diagnosis (*ICD-10^b^*)		
DF20.0 Paranoid schizophrenia	5 (50)	—
DF20.3 Undifferentiated schizophrenia	2 (20)	—
DF20.9 Schizophrenia, unspecified	1 (10)	—
DF22.8 Other persistent delusional disorders	1 (10)	—
DF28 Other nonorganic psychotic disorders	1 (10)	—
Recruitment site		
Capital Region of Denmark	4 (40)	—
North Denmark Region	4 (40)	—
Region of Southern Denmark	2 (20)	—
Level of education		
Primary school	4 (40)	—
Secondary	2 (20)	—
Vocational	2 (20)	—
Further education (<2.5 y)	2 (20)	—
Higher education (>2.5 y)	0 (0)	—
Employment		
Full time or part time	0 (0)	—
Student	1 (10)	—
On sick leave	1 (10)	—
Disability retirement (or retired)	5 (50)	—
Unemployed	3 (30)	—
Duration of voice-hearing (years)	—	16.7 (10.43)^c^
Number of voices		
1	4 (40)	—
2‐5	4 (40)	—
>5	2 (20)	—
Self-reported experience with VR^d^ (range 1‐7; with 1=“beginner” and 7=“expert”)	—	1.3 (1.9)
Months from trial baseline assessment to qualitative interview	—	7.55 (2.73)
PSYRATS-AH-Total^e^ (baseline)	—	33.9 (2.84)
Therapists		
Age (years)	—	46.3 (9.3)
Gender	—	
Women	8 (100)	—
Ethnicity		
White	8 (100)	—
Education		
Psychologist	5 (62.5)	—
Nurse	2 (25)	—
Medical doctor	1 (12.5)	—
Recruitment site		
Capital Region of Denmark	2 (25)	—
North Denmark Region	3 (37.5)	—
Region of Southern Denmark	3 (37.5)	—
Years of professional experience before trial entry		
Working within one’s profession	—	17.1 (9.2)
Working in psychiatry	—	15.1 (7.6)
Working with psychotic disorders	—	13.9 (8.3)
Conducting therapy	—	13.3 (6.9)
Number of challenge therapies commenced	—	13.6 (6.3)

a Not applicable.

b *ICD- 10*: *International Classification of Diseases*.

c Voice-hearing durations used for sampling were based on percentiles from the Challenge trial: 0.05%=1.75 years (n=0), 0.1%=4 years (n=2), 0.25%=7.5 years (n=2), 0.5%=14 years (n=1), 0.75%=20 years (n=2), 0.9%=30 years (n=2), 0.95%=35.5 years (n=1). No trial participants had <2 yrs of AVH (0.05 percentile) and completed their last therapy session within a year, making it impossible to sample from this percentile.

d VR: virtual reality.

e PSYRATS-AH-Total: Psychotic Symptom Rating Scales-Auditory Hallucinations-Total.

### Thematic Analysis

**Figure 1.**
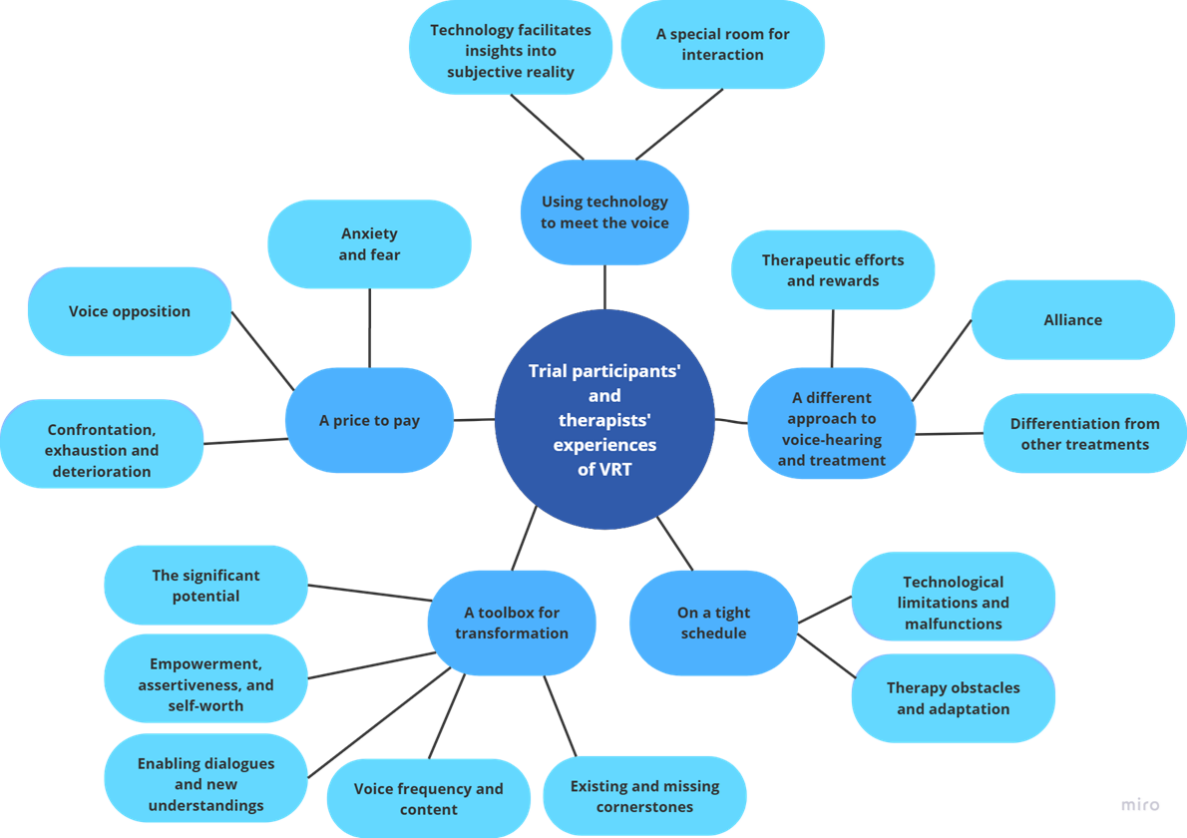
Thematic map illustrating themes and their associated subthemes generated through the thematic analysis of Challenge virtual reality–assisted therapy (VRT) trial participant and therapist interviews.

Thematic analysis resulted in five overarching themes: (1) a different approach to voice-hearing and treatment, (2) using technology to meet the voice, (3) on a tight schedule, (4) a toolbox for transformation, and (5) a price to pay. Within each theme, subthemes were separated out (see Figure 1) with illustrative quotes provided for each (see Table 2).

**Table 2.** Outputs of the thematic analysis, showing themes and subthemes derived from Challenge-virtual reality–assisted therapy (VRT) trial participant and therapist interviews, with illustrative quotes for each.

Theme and subthemes	Illustrative quote—trial participants	Illustrative quote—therapists
Using technology to meet the voice		
Technology facilitates insights into subjective reality	You got a bit more of a tangible experience of it [in VR], than if we had just sat and talked face to face (…) because it was his voice, his image. And I spoke directly to him [the voice] while looking at him. (…) It felt a bit more realistic. [Participant 6]	You’ve got that aspect of observing the dynamic between the patient and the avatar and experiencing it live [in VR]. (…) You definitely get a lot of information that you wouldn’t if we were just sitting across from each other at a desk, looking each other in the eyes. [Therapist 5]
A special room for interaction	It’s strange. For many years, I’ve been unaccustomed to having a dialogue with my voices. But the last two times [in VR], I’ve sat and talked with the voice, with him. I haven’t been able to do so for 20 years. [Participant 8]	I’m well aware that I’m not wearing a VR headset. Still, I feel like I’m in the room. I can follow along on my screen, and I can see what the patient sees. I’m there as the avatar and as myself (…). I have a sense - and often talk with the patients about it - that I’m going in there with them. [Therapist 5]
A different approach to voice-hearing and treatment		
Therapeutic efforts and rewards	You’re working with someone who is actively trying to support you, but at the same time (…) is supposed to sabotage you. It’s kind of strange because the therapist has to be both hero and villain. [Participant 10]	The biggest challenge is figuring out how we can create a change in the [voice] relationship (…). Even though we have a manual, we end up thinking very individually about each person’s history. There are many things to consider. [Therapist 4]
Alliance	[The therapist] is skilled. At putting herself in my shoes. Really listening. Giving input. I have really been able to use it for a lot. Even if it had just been conversations with her, I would still have been able to benefit greatly. But it has just become even better because there was an avatar. [Participant 6]	We were in this together very quickly. (…) We met in a shared work environment, in a shared work project, and it [the alliance] was very easy to establish with the patients. It didn’t take long. [Therapist 1]
Differentiation from other treatments	You just need to take some medication for that, right?' That’s kind of what it [previous treatment] has been like. (…) I’ve talked about what they [the voices] say and so on. But not talked about what I could do to make it better. I think that’s what I’ve gotten here, right? [Participant 6]	Quite a few spontaneously say: ‘It’s nice that there’s someone who wants to listen to it’, or ‘who is so open in asking about the experience’, because it can be something that’s hard to make space for elsewhere. [Therapist 5]
On a tight schedule		
Technological limitations and malfunctions	I think it [reaction to technology problems] depends a lot on how it’s handled. In general, [the therapist] is extremely professional and super sweet. We just continued talking while she tried to get it up and running and she made a joke and such. So, it wasn’t like you got annoyed or angry. It was just delayed a bit. [Participant 2]	(…) There was a glitch in her headset. She could hear what she had said herself, playing on a loop. (…) It probably took 10‐15 seconds before I realized something was wrong. It worsened her voices for a short period after the session: She heard both the voice and herself in a loop. Unfortunately, the next two times, the equipment still didn’t work properly. Not the same issue, but we couldn’t get started at all. [Therapist 7]
Adaptations made	We just had to stop a few times and do some breathing exercises and take off our shoes and get grounded and things like that because it was really, really hard. [Participant 5]	I’ve had a couple of times where it was clear that we couldn’t do it today. Instead, I had to help them with something else. And then schedule a new appointment. There were a few times where I thought it would have been unethical if I had just said, 'You can talk to your case manager about that next week, now we’re putting on the VR headset. [Therapist 8]
A toolbox for transformation		
The significant potential	When he [the voice] says things like: ‘If you don’t swallow a pen’, or ‘if you don’t cut yourself’, or ‘if you don’t overdose, your family will die.’ Then, I go in and say: ‘Isn’t it really you who’s afraid of what might happen to my family?’ ‘Yes, that’s it.’ ‘But are you sure my family will die?’ No, he wasn’t sure about that. In that way, it has helped. [Participant 8]	It [the voice] was her enemy before because it always talked condescendingly, and she had perceived it as if it didn’t want anything good for her. But then it came to her indirectly - what its actual function was - and then she could begin to see it differently and view it as a friend instead. It just needed to express things in a different way. [Therapist 2]
Empowerment, assertiveness, and self-worth	I think a lot of it is having the tools to sort of set them [the voices] straight. I’m not exactly sure if they can understand it, but it’s about the fact that it’s also given me better self-esteem because now I have all these tools for it. [Participant 9]	They [the participants] realize: ‘We’re sitting here openly talking about the voice, the voice doesn’t like it, but nothing happens.’ In some way, the voice’s power diminishes because it becomes less threatening - they realize that the voice can’t do everything it claimed it would. [Therapist 7]
Enabling dialogues and new understandings	It is less aggressive. It has its periods where it can really escalate if it thinks something very dangerous is about to happen. Now, I can almost tell them to calm down. Then, it’s as if it tries to speak a bit more decently. [Participant 4]	When you have that approach that the voice is actually there to help you - it’s just been doing it in the wrong way. That’s something that has made them [the participants] start thinking differently about the voice: ‘Okay, it might always have been discouraging, but it actually did it with good intentions. It just came out the wrong way.’ [Therapist 2]
Voice frequency and content	It’s not so much with insults anymore. Now, we can have a regular dialogue. (...) Instead of calling me ’a big fat idiot’, then ’that might not have been so well done’ or ’you need to do this differently.' It doesn’t have those negative words in it. (…) I can feel that I’m less negative myself as well, so it has a positive effect on me that my voice is less negative. [Participant 3]	It [the voice] has started giving compliments instead, or it has just turned into something like: ‘Remember your glasses when you go out the door’, or ‘you just spoke wrong when you were crocheting’. From being completely critical to suddenly being an extra attention reminder. [Therapist 8]
Existing and missing cornerstones	Unless you’re deeply psychotic, you know that you’re sitting in a hospital and have a therapist sitting right there (…). I mean, she [the therapist] doesn’t stop existing just because I can’t see her. I think, no matter what, you have to accept the premise that this is something we’re playing. [Participant 4]	You are missing a cornerstone in the treatment if you cannot get the patient to buy into the premise. We’ve had someone who said: 'I know that this isn’t real. But if I’m going to get something out of this, I also know that I must pretend that it’s real’. And he benefited greatly from it. It’s fair enough if they say they know it’s not reality. [Therapist 1]
A price to pay		
Anxiety and fear	It was very anxiety-provoking at first. I almost had a panic attack because it was very intense. Or not intense, but it was very overwhelming. To see him [the voice]. [Participant 8]	Patients could be very anxious at the beginning. More than we might have expected. Also about entering VR. We must spend a bit more time at the beginning to go at a pace that people can keep up with. Some are very afraid of their voices. Consequently, we cannot proceed as quickly as in the manual. [Therapist 4]
Voice opposition	It got worse. A lot. Mainly because he [the voice] asked me to stop attending, and I chose to keep talking. I talked to [the therapist] about it and my [regular] therapist in psychiatry, and eventually, I decided to keep going. It definitely affected my daily life quite a bit for a while because it got really bad, and I was hospitalized. [Participant 2]	Well, I have also encountered voices that resort to screaming when they can’t get through with criticizing and make them [participants] stop the therapy (…). We’ve worked with this in VR (…): 'I’ll scream until you stop this therapy. Until you do what I say. [Therapist 4]
Confrontation, exhaustion, and deterioration	I could have some slightly worse days because it could be quite draining, energy-wise, to participate. It could lead to a few negative days. Those are aftereffects of having worked with my voices. The voices could be very negative and macabre. (…) It happens when I’m under pressure. [Participant 3]	She [the participant] experienced that her father [the voice] was holding her in a vice. Literally, and it went on for three weeks. The supervisor and I thought we should stop (…). I didn’t dare to continue, but she wanted to go on. And it ended with the voice disappearing. A fantastic process, but it was definitely intense. [Therapist 3]

### Theme 1: Using Technology to Meet the Voice

VR enabled participants to share the typically private experience of voice-hearing and offered a new way to interact with the voice(s) through the avatar.

#### Technology Facilitates Insights Into Subjective Reality

Several trial participants described giving the voice a face and a body as an essential element of the therapy:

*I desperately wanted something different than a silhouette. Because, I have no control over a silhouette. A face is easier to control. It’s easier to have a conversation with a face than just something floating around.*

[Trial Participant 3]

Some had a clear idea of how the voice(s) looked, while others did not. A few were guided by the voice(s) in designing the avatar, but most experienced that the voice(s) would interfere with the process. For one trial participant, this raised a dilemma: whether to create the avatar based on their own perception or to let the voice dictate its appearance:

*Very quickly I came to terms with the fact that the avatar should be made the way I saw the voice, not the way the voice saw itself.*

[Trial Participant 9]

A high degree of resemblance between the voice and avatar was generally reported, though complete convergence was rare. Most trial participants found some level of similarity essential:

*That makes the premise easier to accept. I wouldn’t be able to accept it at all if it were, like, a little girl sitting there and I was supposed to accept it as my voice. I hear an older man, so it’s not, like, a little baby. That would be completely ridiculous. So, I think it’s extremely important that you’re allowed to design it yourself.*

[Trial Participant 2]

However, some trial participants felt that a less exact match made them feel safer:

*If it resembles too much, then I would just start hearing the voice instead of what* [the therapist] *says. It would be a bit hard to know who’s speaking, if it were completely accurate. So, I felt it was actually a bit comforting that it wasn’t completely accurate during those first times I spoke. But it could have been more accurate in the later sessions.*

[Trial Participant 10]

Achieving insights into what the voice looked like and merely seeing it in front of them was described as helpful or facilitating relief by some trial participants, with one participant mentioning that visualizing it provided a point in space to direct their attention. In some cases, the avatar was found to resemble a known persona from the trial participant’s life, as noted by a therapist:

*I could clearly see who this avatar resembled. Luckily, she [the trial participant] also realized it herself at one point and said, ‘Oh, it’s me. This is a part of me, isn’t it?’ Already at that moment, something shifted for her. It was interesting to witness because I don’t think we would have necessarily reached that realization if we hadn’t been working together on shaping this avatar.*

[Therapist 6]

Through VR, therapists gained insights into the subjective world of voice-hearing, experiencing firsthand how intense, human-like, and overwhelming the voices could feel. It was as if they were granted access to a private, intimate space, allowing them to witness the unfolding dynamics and participants’ real-time reactions—informing their therapeutic approach:

*They are there with their voice, even though they know it’s VR. You get a completely different insight into the subjective world of living with a voice compared to just asking about it.*

[Therapist 4]

Some therapists noted that collaboratively designing the avatar helped trial participants articulate their perceptions, acting as a shortcut to establishing shared understanding. Most trial participants echoed that the VR encounter facilitated a dialogue that was not obtainable in real-world settings:

*It* [VR] *meant that we could somehow get around that therapist-patient barrier, which I think is very clear in psychiatry. Because* [the therapist] *had to take on the role of the voice. This way, she was kind of invited into a world that I would otherwise have a hard time giving her access to. You would never have that kind of dynamic conversation if you were talking about your symptoms. She kind of got to experience them.*

[Trial Participant 2]

Audio recordings allowed trial participants to revisit their responses and progress, with some finding them helpful for reflection. Others shared recordings or avatar images with relatives, peers, or professionals. While this could provoke anxiety about others’ reactions, it also served as a new tool to communicate and explain the inner experience of voice-hearing, fostering shared understanding:

*It’s not just inside my head anymore. It’s kind of out in the open. My loved ones have been let in on what I struggle with.*

[Trial Participant 6]

#### A Special Room for Interaction

VR was seen as creating a distinct therapeutic space, where interacting with the voice through the avatar helped focus attention and reduce distractions. Both trial participants and therapists valued this immersive environment, which offered opportunities for deeper engagement while also presenting challenges:

*It was transgressive* [being in VR], *because suddenly you’re in another place - even though your brain knows you’re sitting in a treatment room that doesn’t look like that. It’s also about giving up control – about not knowing what’s going on around you.*

[Trial Participant 3]

Highlighting the unique experience of creating and interacting with a visual representation of the voice, some trial participants described how the avatar’s tangibility, presence, and direct gaze brought the voice “to life”—as if it were a real person in VR:

*It just became so real. I usually spend a lot of energy thinking, 'No, it’s not real, it’s just in your head.' But then it suddenly felt even more real when it was right there*. (…) *It feels much more real in VR*.

[Trial Participant 5]

Most trial participants did not distinguish between the avatar and the voice. Instead, as reflected in emotional and physical impulses like wanting to hit or confront the avatar, most experienced the interaction as a direct encounter with the voice:

*Due to me being inside this ‘zone bubble’ it was as if we, in fact, sat in front of each other and talked.*

[Trial Participant 6]

However, while some trial participants became fully immersed, forgetting time and surroundings, others remained highly aware of being in the therapist’s office rather than a different world, for example, due to VR graphics:

*It is so different that it feels a bit unreal* (…). *You’re made of flesh and blood… And when you’re wearing the glasses [HMD], it’s like you’re in a cartoon*.

[Trial Participant 1]

On one hand, VR graphics enhanced concentration for some and reassured others that it was not reality, making it less frightening:

*It actually made me feel safe. Because I knew that if something went wrong, it could just be taken away. Click the* [panic] *button if it gets too much. Just take off the glasses [HMD].*

[Trial Participant 10]

On the other hand, some trial participants had to actively “accept the terms,” as the setup or the realism of the interaction did not come naturally to them. Consequently, some approached the intervention with ambivalence:

*When you know it’s not real… but you have to pretend… that it is real, you have to try to… trick your own body. I know that the conversation with the voice is actually just the psychologist. So, I kind of have to try to convince myself that it’s not the psychologist.*

[Trial Participant 10]

Potentially, having to pretend could hinder immersion, yet the awareness that VR was not real also offered reassurance:

*The feeling I get when I can’t distinguish between reality and non-reality - then I get really scared. I didn’t feel that in the same way with this* [VR], *because I could already sense that it wasn’t real.*

[Trial Participant 5]

The mix of structured and unstructured role-play was challenging for some. While lack of scripted lines was anxiety-provoking for a few, others found open dialogue more authentic. VR helped some participants engage more fully, even those initially skeptical, as noted by a therapist:

*That he had to put on the VR glasses [HMD] and engage with what he saw, it just made him go along with the game. Then it was just a completely different dialogue we had.*

[Therapist 8].

### Theme 2: A Different Approach to Voice Hearing and Treatment

This theme highlighted therapists’ efforts and rewards in delivering VRT, the strong therapeutic alliance fostered, and participants’ appreciation for explicitly acknowledging and addressing voice hearing.

#### Therapeutic Efforts and Rewards

Therapists were thoughtful in delivering high-quality VRT, striving to portray authentic avatars, carefully timing VR exposure for optimal impact, and tailoring interventions by connecting symptoms to life experiences—all while upholding high personal expectations of their performance:

*Having to go in and play a role also requires that you can clearly remember what exactly you said last time – where the dialogue ended. So, on days when it wasn’t possible for me to go back and either listen to or read what I had written, I wasn’t entirely satisfied.*

[Therapist 7]

Therapists described VRT’s delivery as a steep learning curve needing thorough training and supervision. Managing avatar dialogue improvisation, monitoring exposure and reactions through the HMD, and adhering to the manual simultaneously was a demanding task. Particularly, reproducing negative voice content in avatar dialogues could be uncomfortable:

*I also found that really difficult at first, because it’s just miles away from how I usually speak to the patients. You would never say those kinds of things to patients in a regular setting. But that’s what it takes for the dialogue to be relevant and believable.*

[Therapist 5]

Remembering the intention of accurately reproducing voice content helped therapists step into the avatar role, though it remained challenging. Direct engagement with the content connected therapists to the reality of the voice, and thereby to the person:

*The harshest voices could cross some of my own boundaries, in terms of what I had to say. Having to say: 'You whore child.' That didn’t roll easily off my tongue, but that was what the voice said. It’s about finding the language that the voice uses. There’s no exposure to it if it’s some kind of light-version. That part hasn’t necessarily gotten any easier*. (…) *But it is a role you’re playing, after all.*

[Therapist 8]

Therapists could feel vigilant and nervous delivering VR dialogues, aware of the intensity and potential risks involved:

*You want to deliver something… really good*.

[Therapist 5]

*Yes, and maybe you also become a little nervous, because I have a sense that you could deliver something that could cause harm, right? Because it’s so intense when you're in there* [in VR]. *There’s a lot at stake in there, so you're on the ball.*

[Therapist 4]

Conversely, therapists were inspired to continue by witnessing participants’ breakthroughs, experiencing a sense of accomplishment and motivation from positive outcomes:

*That’s such empowerment, right? To witness it in the patients is truly fantastic. Once you've experienced it a few times, you get a bit of a taste for it. And think, it’s okay* [reproducing negative voice content].

[Therapist 4]

All therapists reported that delivering VRT demanded extra effort—not only to effectively integrate VR into therapy but also to handle the technology and its malfunctions:

*I think we did it 200 percent and it has worn me out. I also took on a lot of responsibility for the technical aspects as well.*

[Therapist 2]

#### Alliance

Most trial participants and therapists felt the therapeutic alliance formed faster than in usual treatment. Therapists highlighted VR’s unique role in creating a shared experience by referring to the concept of “the common third.” This concept from social pedagogy refers to “an activity or an experience they have together which feels unique in a positive way” [93] or “an arena for equal sharing and participation” [94]:

*It’s a common third: ‘Now we go in here and do this together. Then we go out and talk about the experience.*

[Therapist 4]

Most trial participants echoed the value of the shared activity and collaborative approach in VR, particularly during avatar creation, which was often a powerful experience:

*The way you collaborate, that’s what works for me-doing it together. If I had made the avatar myself, I’m not sure I would have gotten as far.*

[Trial Participant 3]

Therapists were key in enabling shared experiences, with their role-shifting, guidance, and support during avatar dialogues seen as essential to therapeutic collaboration. While some trial participants felt self-conscious speaking to an avatar controlled by a nearby therapist, others found the therapist’s dual role comforting but odd:

*The fact that she* [the therapist] *was both there as a challenger and a support, was strange, but in a way reassuring to know that the person who is challenging me right now is also one of my biggest supporters throughout this process.*

[Trial Participant 9]

While participants valued common therapeutic factors like the therapist’s qualities, VR appeared to add a distinct dimension to the alliance. It fostered a deeper, shared understanding between therapist and participant—beyond what could be attributed to therapist skill or receiving a novel talk therapy alone:

*It* [using VR] *meant a lot. That she* [the therapist] *kind of got to have a bit more of a real image… a dynamic image… of the world that I carry with me. That it wasn’t just me sitting there, having to talk about what had happened since last time, but that we could, in a way, create what was happening together.*

[Trial Participant 2]

The combination of VRT’s shared experiences and common therapeutic factors made trial participants feel safe and confident in the therapist—marking a notable contrast to some participants’ prior experiences with psychiatric care:

*There was just some kind of respect and understanding that I haven’t had elsewhere. I’ve had treatment providers with 25 years of experience, but they had no idea how it was like* [hearing voices].

[Trial Participant 4]

#### Differentiation From Other Treatments

For trial participants, the main motivation for joining the Challenge trial was the desire for an alternative to medication—driven by past experiences of limited effectiveness, intolerable side effects, or a preference for gaining tools to manage voices rather than focusing solely on “symptom treatment.” Most trial participants felt they worked alongside the therapist to understand and manage the voice(s), valuing being treated as more than “just a number.” One therapist recalled a participant finding the participatory and active nature of VRT helpful:

*It’s not just about adjusting medication. That’s more something others do to him* [the trial participant]. *But if he gets something he can use and work with himself, then he gains a sense of control.*

[Therapist 8]

Unlike previous experiences, trial participants appreciated the explicit focus on voice-hearing and found avatar dialogues to be a novel, tangible way to engage with the voice(s). By externalizing a voice, participants and therapists were able to engage with it collaboratively, in contrast to earlier treatments positioning the voice(s) as a problem within the individual. Many valued that the voice(s) were treated as real experiences rather than merely symptoms to eliminate:

*For the first time ever, it* [the voice] *has been treated as if it were real. Psychiatry is really good at immediately saying, ‘But it’s just a symptom of your schizophrenia.’ And then approaching it from that perspective.*

[Trial Participant 2]

Therapists echoed the positives to focusing on voice-hearing and exploring it in VR, which provided structure, a shared goal, and rapid insight into participants’ experiences. They were impressed by participants’ ability to stay engaged despite challenges. Many participants expressed relief and satisfaction at finally being asked in-depth about the voice(s)—something they felt was often overlooked in other treatments. While sharing voice-hearing experiences could feel transgressive for some, therapists noted that VRT often made such disclosure easier, helping to normalize the experience. For some trial participants, this openness reduced feelings of shame or self-stigma:

*What this therapy has taught me is quite a lot, because I know I’m not the only one who has been part of it. All of those who have taken part… they all hear voices. It’s not just me. That has made it less of a taboo for me to talk about it.*

[Trial Participant 9]

### Theme 3: On a Tight Schedule

This theme explored technological challenges and the adaptations therapists made during therapy.

#### Technological Limitations and Malfunctions

Trial participants and therapists noted that avatar design limitations—including lack of options for attire, hairstyles, body shapes, or nonhuman features—sometimes reduced how well avatars represented the voice. As a result, therapists occasionally had to adapt planned dialogues to fit the available designs. Besides these limitations, therapists faced challenges learning and navigating the hardware and software, compounded by frequent technical errors:

*There have been really, really many technical problems. It has been a strong presence for all the therapists. It has disrupted therapy incredibly much*. (…) *When there’s no sound, or no image, or something is wrong - then we can’t do it.*

[Therapist 4]

Therapists found the technology malfunctions frustrating, especially when they left participants distressed or prevented crucial exposure work:

*All the technical stuff, I would almost say, that was probably the hardest part. Because everything was so unfamiliar to us. And we didn’t have anyone right next to us to help.*

[Therapist 2]

Despite therapists’ frustration, most interviewed trial participants seemed unaffected by technical issues, with only one finding them stressful. Therapists noted trial participants and relatives were generally understanding, viewing errors as part of a new treatment. Some therapists even saw these moments as opportunities to build rapport, occasionally reversing roles as trial participants helped with the technology, fostering a relaxed atmosphere.

*It’s a bit annoying when it doesn’t work right away. Which technology sometimes doesn’t do. I mean, sometimes we went half an hour over time. Because the equipment just had its problems every now and then. That didn’t really matter to me, to be honest.*

[Trial Participant 2]

#### Therapy Obstacles and Adaptations

Therapists valued the short therapy format for boosting participants’ motivation and adherence but often found the schedule too tight. Delays from technical issues, participant needs, and organizational demands required frequent adaptations. While the manual provided helpful structure, it lacked specific guidance for complex cases, prompting creative solutions and sessions to run over time. Such complexities often included high anxiety, delusions, voice dynamics, or unresolved trauma:

*I remember a case with a lot of traumas. After seven sessions, it just felt really short, because we couldn’t get through it all, and the voice was very much connected to the trauma. She* [the trial participant] *would fall back into these traumas when the voice said things related to the trauma. It was also a person from those traumas that she heard speaking to her. I really wish I had had more time.*

[Therapist 4]

Furthermore, organizational constraints sometimes limited therapists’ ability to deliver optimal VRT. Competing tasks and multiple cases led to compromises in preparation, with some therapists working after hours to stay ready. The lack of scheduled buffer time for technical issues caused sessions to run over, which a trial participant accepted but noted may be unfeasible in standard care due to stricter scheduling (Participant 2).

### Theme 4: A Toolbox for Change

This theme highlighted the changes experienced and reflections on therapeutic targets, mechanisms, and prerequisites influencing outcomes.

#### The Significant Potential

Although not all trial participants experienced improvement, therapists were optimistic about the therapy’s potential, with one calling it “revolutionary” and “life-changing” for some. A trial participant expressed a similar sentiment:

*I used to lie there when going to sleep, thinking: ‘I really hope I don’t wake up again’. Now, it’s completely the opposite. Now, I go to bed looking forward to waking up the next day.*

[Trial Participant 4]

Despite some participants feeling ambivalent about changing or letting go of the voice(s), fearing what the future might bring, most welcomed the changes:

*I can feel that I have a better life now*. (…) *I can go shopping more easily and I can go out and walk and stuff.*

[Trial Participant 7]

However, some therapists expressed disappointment over measurements not being able to detect changes achieved through VRT, including improved communication with the voice, brief relief, or shifts in tone or content. VRT also brought subtle or indirect changes, including increased energy, enabling participants to pursue goals like obtaining a driver’s license, starting school, or building a family:

*Things happen - maybe one voice among several disappears, or subtle shifts occur in what the person can now decide for themselves, something they couldn’t before. These changes can be really difficult to capture.*

[Therapist 5]

#### Empowerment, Assertiveness, and Self-Worth

Assertive communication, boundary-setting, and disrupting the power balance helped trial participants regain control, a change therapists found moving. Most felt more equal or superior to the voice—an unusual shift sometimes met with fear of setbacks. Some became less fearful, more accepting, or able to negotiate with the voices, leading for some to improved relationships or self-worth:

*I have become more confident in myself because, before, one of the things I was really hard on myself about was that I couldn’t figure out how to stand my ground. But through the process* [VRT]*, I’ve learned that I can do that.*

[Trial Participant 9]

Recognizing that the voice is neither dangerous nor powerful may help reduce anxiety, as a trial participant suggested:

*As I’ve become less afraid, his power has also diminished. Just automatically*. (…) *Because as I’m less afraid of him, then I get less upset when he says mean things. So, he has less power to make me sad*.

[Trial Participant 2]

In trauma-related cases, a therapist highlighted the usefulness for the avatar to say new, constructive things in VR—potentially improving the relationship, if applied to the voice:

*I experience, when we talk about trauma, that it* [VR] *really can make a difference. Having the avatar specifically say some of these things, like, 'It was me who was completely wrong about that. And not you.’ Or, 'I don't have that power today. I can see that now.' To separate the past from the present: 'I was a big grown man back then, and you were a little girl. You couldn’t do anything. So, I had the power then.*

[Therapist 4]

#### Enabling Dialogues and New Understandings

Many participants saw the ability to engage in dialogue with the avatar—and to transfer this to the voice(s)—as a key outcome of therapy, transforming it from an unresponsive, 1-way communication:

*Before, it was just those same sentences they used all the time, and no matter what I answered, it would just be degrading. Now, we can actually sometimes have a conversation; with some of them I can talk.*

[Trial Participant 4]

Besides enabling dialogue, therapists aimed, when feasible, to foster a new understanding of the voice and its intentions:

*Maybe it warns me when I’m in situations that are hard to handle. I mean, it’s about getting a different understanding of what it stems from.*

[Therapist 6]

Some trial participants began to view the voice(s) as having positive intentions but poor communication skills, making them feel more human-like. This shift helped some disengage from the demeaning messages and adopt more accepting, compassionate responses, improving their relationship. However, not all participants embraced this more positive interpretation, as a therapist noted:

*I've also had several patients who have said that they simply can't see that there could be any meaning to it. They're really angry at their voice because it has ruined their whole life, and they can't see anything positive about it* (…) *and that’s something you have to acknowledge.*

[Therapist 3]

#### Voice Frequency and Content

Some trial participants reported a reduction in voice frequency to varying degrees, with fluctuations occurring within the same person, ranging from minutes to hours, depending on the day:

*Now I’m getting used to it a bit, right? But at first, I thought there must be something completely crazy going on, since there were no voices—that I had been killed or something like that—but there wasn’t.*

[Trial Participant 8]

Therapists observed changes in voice content, including voices asking for participants’ opinions, becoming less self-assured, adopting a neutral, observational tone, or even becoming more positive. Some trial participants echoed such changes:

*They might say, ‘Oh, I think that guy is spying on you and keeping an eye on you.’ And then they ask, ‘What do you think?’ I’d say: ‘I don’t think anyone is really trying to watch me.’ ‘Oh no, they could actually see that.’*

[Trial Participant 8]

#### Existing and Missing Cornerstones

Therapists reflected on factors influencing therapeutic outcomes, noting that some trial participants saw no improvement, especially when voices lacked a relational aspect or power dynamic, making them harder to engage or shift through therapy:

*It* [the voice] *was like continuing in the same vein, with three fixed phrases. We just couldn’t get it to stop. It might also be me, maybe I approached it the wrong way, but it felt very stuck to me*

[Therapist 4].

Barriers perceived by therapists included participants reluctant to disclose voice experiences, lack of emotional reactions, or difficulties with (voice) presence. Untreated trauma and delusions could sometimes impair progress. However, those individuals that therapists judged to have more limited cognitive ability seemed to do just as well as those judged to be of higher intelligence.

Therapists suggested that positive outcomes were more likely when trial participants showed inner motivation, persistence through challenges, and readiness to form a new relationship with the voice(s). Factors like being further along in recovery, support from others, completing homework, and making life changes outside therapy also contributed. Especially, therapists viewed engagement, (voice) presence, and immersion as deeply interconnected and essential to the therapeutic process:

*I think immersion already begins when we create the voice, right? That they engage, that they relate to things like eye color - but if they just say, ‘just pick one,’ or something like that. That always signals a little bit of a bad start, right? In terms of their immersion*

[Therapist 8].

Multiple factors were suggested by both trial participants and therapists to influence presence. Beyond graphics, a trial participant highlighted their therapist’s endeavors:

*If it was something we had talked about three sessions ago–she* [the therapist] *could just make a little, like, move, to bring it in. And then she could create some kind of complete picture of him* [the voice]*, which made it easier for me to believe in the premise*

[Trial Participant 2].

### Theme 5: A Price to Pay

This theme highlights the adverse effects during and between sessions, including anxiety, negative voice reactions, exhaustion, and general symptom worsening.

#### Anxiety and Fear

The first avatar encounter was intense and anxiety-provoking for most trial participants, triggering strong emotions (eg, anger, crying, or panic) and physical reactions (eg, nausea, hypertension, sweating, or flight). Therapists were often surprised by the intensity of these responses, which, despite participants being informed that anxiety was expected, at times led to emotional flooding and required adaptations or delays.

*When they enter VR for the first time and have to meet the avatar, I think many of them react strongly. The exposure is really, really high, so you can clearly see that this is really uncomfortable*.

[Therapist 6]

Most trial participants echoed that the first encounters in the initial phase could provoke anxiety:

*The closer it* [the avatar] *came, the more I couldn’t control what was happening inside me. I became furious, angry, and sad all at once. And anxious. Very anxious. Panicked. I was shaking. I collapsed in the chair. It was either run or fight.*

[Participant 4]

One trial participant ascribed the anxiety to a loss of control and the unaccustomed situation:

*In the beginning, it caused anxiety. Because a bit of my control was taken from me*. (…) *Suddenly, you're sitting face-to-face. I don't normally do that with my voice.*

[Trial Participant 3]

Even trial participants familiar with VR reacted to avatar dialogues with emotions like “shock and surprise” (Trial Participant 10) or “sadness and anger” resulting in using the panic button (Trial Participant 9). This suggests that it may not be VR per se, but the specific way it is applied that elicits such reactions, as one therapist reflected:

*It* [VR] *really does something to some of the patients. It’s almost like they tear the glasses off the first time. So, it’s really a powerful tool to be able to use.*

[Therapist 2]

Therapists viewed co-presence in the room as essential for providing timely support and ensuring safety during anxiety. The immersive format did not seem to disrupt the therapeutic bond; in fact, trial participants valued the therapist’s physical presence as reassuring, with a participant noting it helped them avoid feeling “trapped” in VR:

*If I had been sitting there all alone, I think it would have been really difficult for me to handle. But when* [the therapist] *was there and my contact person was there, it was a bit easier to handle the anxiety and all the emotions that came up while sitting inside* [VR].

[Trial Participant 8]

#### Voice Opposition

Most trial participants reported that the voice(s) reacted negatively to their involvement in the therapy—some dismissing it, urging withdrawal, or becoming angry or condescending. Others expressed skepticism, resisted the avatar design process, or disrupted the VR dialogues:

*It’s fraud and nonsense’; ‘You can't use it for anything’; ‘It’s just people who want to play with you; ‘They don't want anything good for you’; ‘You'll never get rid of us.’ It’s been things like this all the way through. They [the voices] really tried to talk it [VRT participation] down so I wouldn't believe it.*

[Trial Participant 6]

Voices interfering could complicate therapy:

*The therapist might tell the avatar to be kinder to me, but the voice might say, ‘I don’t want to,’ at the same time. I find it really confusing.*

[Trial Participant 5]

Standing up to the voice could have consequences. A trial participant questioned whether posttherapy hospitalizations were linked to VRT, while another required hospitalization during therapy but later resumed and completed the sessions:

*At first, he* [the voice] *was totally indifferent. He thought it was a ridiculous trial and that it was a waste of both our time: ‘But if that was what I wanted to do, then I should just do it, because I always did what I wanted.’ But then, during therapy, he didn’t want me to show up anymore and became very threatening and negative. This eventually led to me being hospitalized.*

[Trial Participant 2]

Despite interference or opposition from voices, trial participants generally stood up to the voices and continued therapy:

*I fought against it, at least. I said, ‘Now. I have to get through this.” Then I told the voices, 'Now you need to be quiet.*

[Trial Participant 7]

#### Confrontation, Exhaustion, and Deterioration

As the avatar encounters could be demanding, some trial participants were exhausted afterward:

*After each session, I had to go home and lie in bed for half an hour to recover. Because it was really transgressive to go in and talk to the voice in VR and work with it. It takes energy.*

[Trial Participant 10]

Confronting the avatar often led to postsession exhaustion, with responses ranging from sleepiness to hyperarousal, insomnia, or worsened symptoms. Some trial participants had experienced similar responses to past stressors:

*There’s always a small price to pay afterward, and that’s just how it is*. (…) *Fatigue and lack of energy, along with increased symptom activity*. (…) *When I’m stressed, that comes.*

[Trial Participant 5]

Although both therapists and participants were familiar with symptoms worsening from new activities, VRT seemed to carry an additional cost for some. Though already accustomed to using medication as a coping strategy, a participant took sedatives after each session, on advice from nontrial staff, to manage anticipated symptom flare-ups. The intensity and confrontational style of VRT were seen by some therapists as particularly taxing, affecting both participants and therapists alike.

*You're putting much more pressure on the patient than you do with other treatment methods. You can also clearly see that the patient gets worse*. (…) *That’s one of the burdens as a therapist*.

[Therapist 1]

Therapists identified the intensity of VRT as the primary reason for dropout, with some participants opting for less confrontational methods to manage the voice(s):

*It was too confrontational. The person preferred to adjust their medication rather than being in such a therapeutic process. They thought it was simply too intense.*

[Therapist 5]

## Discussion

### Principal Findings

This study explored 10 trial participants’ and 8 therapists’ experiences with VRT within the Challenge RCT, generating five overarching themes: (1) using technology to meet the voice, (2) a different approach to voice-hearing and treatment, (3) on a tight schedule, (4) a toolbox for transformation, and (5) a price to pay. Findings share several similarities with qualitative research on AT [57,67], RT [23,24], and TwV [27,28,95], as elaborated below.

A total of 3 implementation outcomes (acceptability, appropriateness, and feasibility) are used to interpret and contextualize findings beyond the RCT. Following conceptual distinctions [56], acceptability is perceived satisfaction with VRT in terms of content, complexity, and comfort; appropriateness is VRT usefulness and relevance; and feasibility is perceptions of whether VRT could be conducted in practice, considering enablers and barriers. Table 3 summarizes the main learnings and implications.

**Table 3.** Summary of discussion points highlighting the main findings of identified themes within implementation outcomes (acceptability, appropriateness, and feasibility) and their clinical implications.

Implementation outcome	Findings	Clinical implications
Acceptability		
Content	(+)^a^ Acknowledging approach. (+) VR:^b^ Embodiment and externalization. (+) VR: Meaningful space for dialogue. (-)^c^ Suboptimal avatar design. (-) Suboptimal (voice) presence. (-) Discomfort reproducing content. (+) Deeper insight into voice-hearing. (+) Strong alliance.	Voice(s) can be acknowledged as real phenomena, not symptoms. Voice(s) can be externalized and made more tangible in VR. Voice(s) can be dialogued with as avatar(s) in VR. Voice(s) may have highly specific appearance(s). To some, challenges remain with establishing (voice) presence in VR. Reproducing verbatim content is hard, but necessary and meaningful Avatar dialogues may deepen therapists’ understanding of voices. Shared activity or “common third” may support alliance-building.
Comfort	(-) Malfunctions, disruptive and frustrating. (-) Anxiety, exhaustion, and deterioration. (-) Voice reactions.	Operational reliability of software and hardware should be ensured. Individualized starting point and continuous tolerability monitoring. Voice(s) may oppose therapy.
Complexity	(-) Demanding therapy for participants. (-) Role-play and interfering voices. (-) Nontraditional therapeutic skills.	Attention should be given to introduction and monitoring. Consider direct voice communication to create space for therapy. Training, supervision, and standardized materials are essential.
Appropriateness		
Usefulness	(+) Positive changes. (-) Measurement insensitivity to changes. (+) Individual means and targets. (+) Sharing avatar picture or recording.	Changes can be transformative, but also subtle and multifaceted. Individualized meaningful outcomes could be discussed. Treatment focus should be individualized. Encourage sharing to facilitate normalization and reduce stigma.
Relevance	(+) Embodiment and externalization. (+) Avatar dialogues. (-) Assertiveness versus compassion.	Voice(s) are more tangible when given a face. VR-supported assertiveness practice. Flexibility in whether compassion comes from therapist or avatar.
Feasibility		
—^d^	(+) Therapists’ motivation and efforts. (+) Participants’ motivation and resilience. (-) Participants’ engagement and reactions. (-) Limited organization resources. (-) Technological malfunctions.	VRT^e^ requires dedicated therapists as many elements will be new. Consider whether the timing is right. Sensitivity for optimal exposure. Organizational arrangements should allow sufficient time for therapy delivery, including handling of diversions or unexpected disruptions.

a (+): positive aspect of virtual reality-assisted therapy (VRT).

b VR: virtual reality.

c (-): area of concern.

d Not applicable.

e VRT: Virtual reality–assisted therapy.

### Acceptability

Areas of high acceptance included the novel approach to working with voices, the specific focus on voice-hearing, and the collaborative relationship (themes 1‐2). Contrastingly, technology malfunctions, limited resources, and participant discomfort were areas of concern (themes 3 and 5).

### Content

#### The Acknowledging Approach

Trial participants appreciated that voices were acknowledged and approached as a real phenomenon rather than mere symptoms requiring medication (theme 2). This resonated with their motivation to seek alternatives to antipsychotics and reflects the broader tendency of preferring psychological therapies over pharmacological treatments [96]. It also echoes findings from TwV, where discouragement with previous treatment outcomes was highlighted as a key motivator for engagement [28]. Therapists’ acknowledgment of voices may serve as an important first step in reducing stigma, counteracting perceived stigma from mental health providers that can “get under the skin” of service users and contribute to disempowerment [97]. The acknowledging approach was seen as trust-generating (theme 2) and mirrors the central normalizing tenet within the Hearing Voices Movement [16]. RT and TwV approach the voice(s) similarly, though typically without AT’s and VRT’s confrontational style [18]. Highlighting this commonality is important, as it supports the case for implementing a broader range of talk-based therapies for voices in routine practice, where dialogical engagement with voices remains relatively uncommon [98].

#### VR Affordances, Avatar Features, and Presence

VR was valued for creating a unique space to interact with voices (theme 1), echoing findings that VR can offer a meaningful environment for practicing therapeutic techniques, such as in agoraphobia treatment [66] or mindfulness practice in mood and anxiety disorders [99]. Similarly, VR-CBTp for paranoia supports direct engagement with paranoia-eliciting scenarios and affords greater exposure time than standard CBTp [100]. Trial participants emphasized the need for avatars to be realistic enough for believability, but not so lifelike as to threaten their sense of safety (theme 1). This aligns with research suggesting that cognitive awareness of VR’s unreality can facilitate engagement [101,102] and help maintain a sense of safety, even when physical reactions occur [66]. Likewise, AT accounts show that avatar accuracy influences initial reactions, ranging from anxiety to disconnection, with discontinuation attributed to lack of realism [67]. While therapists identified (voice) presence as crucial for engagement and outcomes (theme 4), secondary Challenge RCT analyses showed that the sense of (spatial) presence was unrelated to outcome [44]. This underscores the need for clearer presence terminology and sensitivity in measurement [37], as well as further investigation into potential interaction effects, such as anxiety reduction [41]. Some trial participants felt they were meeting the voice itself in VR (theme 1), reflecting findings on presence and avatar realism in other studies [41,46,57]. Others struggled with (voice) presence or connecting the avatar to the voice (theme 4), mirroring findings from AT [67]. Given the bidirectional relationship between emotions and presence [38,39], and the role of (voice) presence in AT outcomes [41], VRT delivery must balance optimizing (voice) presence with avoiding excessive anxiety [40] to optimize outcomes.

#### Reproducing Voice Content

Therapists felt discomfort reproducing negative voice content but recognized its importance in avatar authenticity (theme 2), empowering trial participants to reclaim disempowering words [29]. Similarly, in a study on attitudes toward voice simulation training, therapists noted that while authentic simulations could cause distress, they were viewed as essential to preserve authenticity and avoid trivializing the experience [103]. Without proper hands-on training, this discomfort could lead to avoidance in routine care. The broader literature suggests that training in exposure techniques is associated with greater use of exposure therapy [104], and that therapist anxiety can cause clinicians to avoid or minimize treatments that elicit anxiety in both patients and themselves [105]. Difficulty and discomfort with reproducing negative voice content has been reported across AT [67], RT [24], and TwV [28]. In VRT and AT, this task falls primarily on the therapist, in TwV it rests with the hearer, and in RT, it may be shared between both through role-plays. This distinction is important, as responsibility for reproducing voice content may influence the therapy’s acceptability for those tasked with carrying it out. Furthermore, TwV findings show that dialogical work can burden both participants, due to trauma’s aftermath, and therapists, who bear witness to it [95], which highlights the need for extra care for both parties. Ensuring an open, nonjudgmental approach to all voice content and obtaining informed consent about what is used are therefore crucial considerations for future implementation.

#### Deeper Insight Into Voice-Hearing

Therapists appreciated the structured intervention (theme 2), but creative adaptations (theme 3) were often relevant to maintain a degree of individual flexibility. This underscores the importance of a flexible, formulation-driven approach aligned with individual needs [106-108] and preferred treatment outcomes [109]. Therapists also valued the deeper insight into voice-hearing offered by the intervention (themes 1‐2), mirroring AT participants identifying how therapists developed a distinct depth of understanding via the avatar that could make them become an ally [67]. Given the distinction and divide between therapists’ second-hand clinical knowledge and first-hand lived experience of voices [103], avatar dialogues may help bridge the gap, thereby deepening therapists’ subjective understanding of voices. In this way, VRT could function both as a gateway for targeted intervention [34] and as a form of professional skills training, similar to voice simulation training [110].

#### Therapeutic Alliance

Trial participants and therapists found VRT rapidly fostered a strong therapeutic alliance (theme 2), echoing findings from AT [57,67]. A similar observation has been reported in TvW, where the therapists describe voice work as having a distinct intimate quality [95], and participants emphasize the importance of the facilitator’s personal qualities [27,28]. Some trial participants contrasted the trusting therapeutic relationship to past treatment experiences (theme 2), mirroring AT participants highlighting the importance of core therapeutic skills and person-centered flexibility [67]. As VR supported a process of joint exploration and collaboration (theme 1), it may enhance alliance-building by enabling shared engagement through a tangible, visible representation of the voice—what therapists described as a “common third” [93,94]. Given the therapeutic alliance’s established links to engagement and symptom reduction [111], but mixed evidence regarding collaboration or formulation [112], further research is warranted into VR’s potential to enhance alliance. Similarly, other research has shown that using smartphones to record voice-hearing experiences can accelerate rapport-building [113], suggesting that digital technologies can enhance therapeutic relationships by making internal experiences more tangible and communicable.

### Comfort

#### Frustrating Malfunctions

Therapists found technology malfunctions to be highly disruptive (theme 3), occurring in 48% of Challenge RCT therapies [43]. While trial participants were largely unaffected, consistent with AT reports [29], therapists experienced emotional strain and increased workload. These issues may hinder implementation as they confirm clinicians’ concerns that VR increases time and workload demands [69], compounding job stressors to the already demanding context of emotional labor [114,115].

#### Anxiety

As expected in exposure-based therapy, emotional reactions and symptom increases were common, particularly during early avatar encounters (theme 5). Trial participants reported a range of emotional responses (themes 1, 4, and 5), consistent with previous research [45,47]. More positive and less negative feelings were reported as therapy progressed, mirroring other findings [46]. Initial avatar encounters, however, could trigger anxiety (theme 5), likely contributing to the 29% of Challenge RCT therapies requiring exposure adjustments and most withdrawals occurring after the first VR session [43]. Withdrawal in the initial confronting phase has been observed across VRT [49] and AT [29], with emotional experiences becoming temporarily more difficult [67]. In TwV, hearers and voices emphasized the need for a “safe base” for dialogue work, as the process was anxiety-provoking and required trust and timing [27,28]. Anxiety tends to decrease across AT [41] and VRT [46,49], and 79% of Challenge RCT participants completed all sessions [43]. This indicates general tolerability, consistent with AT findings where emotional intensity of high voice presence was well tolerated [41,67]. VR may intensify expected anxiety (theme 5), highlighting the importance of finding a “sweet spot” where anxiety is neither too low nor overwhelming [66]. Establishing an appropriate starting point and allowing adequate time for preparation, monitoring, and calibration appears essential for optimizing outcomes [40].

#### Voice Reactions

Most trial participants reported negative voice reactions (theme 5). In Challenge RCT, voice worsening peaked at session three, with 37% reporting it [43], suggesting early but transient symptom exacerbation similar to trauma-focused treatments [52]. However, a trial participant attributed a hospitalization to increased voice severity (theme 5) but later resumed and completed therapy. Some participants linked worsening to general stress or the initiation of new treatments (theme 5), consistent with previous research [116,117]. Compared to a VR anxiety trial in which only 5% reported voice worsening [118], voice reactions may depend on the therapeutic application of VR. Notably, only a small AT subgroup experienced voice worsening [29], mirroring participants’ own perspectives [57] with one noting increased aggression of voices only after completing therapy [67].

### Complexity

#### Demanding Therapy

Some trial participants found VRT cognitively and emotionally demanding (theme 5), reflecting the intense emotional expressions often encountered in AT [45] and mirroring accounts from TwV highlighting the intensive nature of engaging with voices [28]. The emotional toll of avatar dialogues has even been presented as a reason for dropout in AT [67].

#### Nontraditional Skills

Therapists struggled to juggle technology, manual adherence, voice-content reproduction, and exposure monitoring (themes 2‐3). They also needed to draw on complex, nontraditional skills to deliver avatar dialogues, consistent with previous descriptions of the demanding, “actor-like” approach [20,29,49]. While therapists reported gaining confidence and skills over time, particularly by building a repertoire of responses for avatar dialogues, they described VRT delivery as a steep learning curve (theme 2). Importantly, this was despite their high levels of qualification and psychiatric experience (see Table 1). Echoing research on the varied techniques used in avatar interactions [119], this underscores that therapeutic competency in VRT requires more than a “plug and play” approach. Supporting therapist confidence in working with voice-hearing—through supervision [120] and standardized materials [121]—should be central to future implementation efforts. Specifically, it seems warranted to incorporate structured role-plays and simulation-based training into both initial preparation and ongoing supervision, as also noted in TwV [95].

### Appropriateness

Most trial participants and all therapists viewed VRT as a useful and relevant way of working with voices, indicating high appropriateness.

### Usefulness

#### Positive Changes

Most trial participants, supported by the therapists’ observations, reported gaining tools to manage voices or noted positive changes like increased control or new ways of relating to the voice(s) (theme 4). This echoes therapeutic targets [29] and accounts of positive shifts in voices in AT [67], voice dialogue as a powerful enabler of positive change in TwV [27], and changes within the individual, the voice, or their communication observed in RT [23,24]. Therapists noted that subtle but significant changes—like new opportunities for dialogue with voices or shifts in their content or frequency—could be missed by standard assessments (theme 4). This highlights the need for more sensitive measures [14], as even minor changes can be life-changing, illustrated by a VRT case experiencing 15 minutes daily without voices [122] and the diverse ways resources for moving forward can manifest following TwV [28]. However, voices could remain distressing or unchanged (themes 4‐5)—a point also reflected in TwV, where some changes were constrained by the lasting impact of past adversity [28]. In AT, most voices persisted, but their impact was substantially reduced for many [67], prompting debate about what constitutes a successful outcome [14]. Future research should explore whether digital approaches like VRT and AT produce similar or distinct outcomes [123,124], and whether they enhance sharing, to better assess the cost–benefit of investing in such technologies.

#### Individual Means and Targets

Most trial participants experienced changes in voice communication that extended somewhat into daily life (theme 4), mirroring shifts in communication between participants and avatars during therapy [96] but also difficulties translating changes to voices in AT [67]. Some trial participants continued practicing assertive communication with the voice(s), while others focused on self-understanding or making sense of the voice(s) (theme 4). This supports the exploration of AT in both brief and extended versions [30,58], tailoring the intervention to individual needs and therapeutic targets [29]. It also underscores the need to study which therapeutic approaches work best for different people at different times, since a poor fit can hinder engagement, as indicated in AT [67]. For example, whether exerting power over voices or fostering greater understanding and acceptance proves more helpful may inform decisions on which relational therapy is best suited to a given voice-hearer, as noted elsewhere [27].

#### Sharing Avatar Picture or Recording

Most trial participants valued sharing the avatar picture or audio recording to help others understand how they experience voice-hearing (theme 1). In Challenge RCT, 77% had used either material, 65% had shared the picture, and 41% had shared the audio recording [43]. Sharing could foster feelings of acknowledgment or normalization (theme 2), extending previous findings showing satisfaction with using AT recordings [57]. This is significant given the high prevalence and detrimental effects of stigma in this population, as well as the difficulties addressing it [125-127]. Sharing is not unique to VRT or AT as it also occurs and brings a sense of relief in nondigital therapies like RT [23,24]. However, digital tools may have an advantage, as experiences of hearing voices can be shared concretely through images or audio (theme 1) and, thus, help support living openly with voice-hearing without the burden of shame or stigma [16].

### Relevance

#### Embodiment and Externalization

Most trial participants and all therapists considered VRT a relevant way to work with voices (themes 1‐2). Particularly, dialoguing with the avatar as a proxy for the voice was valued, echoing the appreciation of direct voice dialogue in TwV [27]. Trial participants valued the avatar’s embodiment of the voice, which offered a tangible and direct means of communication (theme 1). “Giving the voice a face” was seen as powerful by some, highlighting the value of externalizing an internal experience. This feature is central to AT [20], where focusing on the avatar’s face has been shown to enhance assertiveness by allowing participants to direct communication toward something specific and externalized [67]. However, it contrasts with direct dialogue work in TwV and voice role-plays in RT. In RT, role-playing a voice can be challenging to some because it requires uniting voice and body [24], which is the opposite of the externalization in VRT or AT. However, limitations in avatar design (theme 3) sometimes made it harder for trial participants to relate to the avatar as they would to the voice, a challenge also noted in AT [67]. This finding aligns with previous research linking greater voice characterization to increased behavioral engagement [128] but contrasts with secondary Challenge RCT analyses indicating that auditory and visual resemblance of avatars shows only weak, non-significant trends toward reducing voice frequency [44].

#### Avatar Dialogues

Therapists found assertiveness and self-worth training highly relevant and were encouraged by participant progress, reflecting findings of changing avatar interactions [47] and increased positive emotions during interactions [46]. However, therapists noted that missing cornerstones (eg, suboptimal (voice) presence or life instability) could render the dialogical approach less relevant (theme 4), mirroring findings from AT that its suitability depends on the mental state and the stage of recovery [67]. Therapists observed that participants often showed remarkable resilience during avatar dialogues, which may go unnoticed in other settings but is likely supported by the context [29], and used this observed strength to empower participants in standing up to the voice(s). This aligns with the AT participants’ perspectives, where determination and gradual improvements facilitated engagement [67]. Likewise, most trial participants found avatar dialogues useful for practicing assertiveness (theme 4), despite sometimes feeling confused, anxious, or awkward, similar to role-play experiences in RT [24]. Notably, some trial participants and therapists experienced that shifting from assertiveness to compassion toward the voice could be inappropriate given its negative impact (theme 3). Therapists were mindful of this tension and carefully weighed when to encourage compassion. As compassion-focused therapies for distressing voices are emerging [129,130], further research is needed on how best to navigate such ambivalence or tension that might arise from compassion blocks [131,132] or trauma-informed frameworks [133,134], e.g., when the voice represents a former perpetrator. Clarifying which therapeutic elements can be flexibly adapted while maintaining manual fidelity is key to guiding implementation and tailoring therapy to individual preferences [135].

### Feasibility

#### Therapist Barriers

Therapists emphasized that thorough training, feedback, and supervision were essential for feasible VRT delivery (themes 2‐4), echoing research pointing to the multifaceted nature of AT requiring experienced therapists [29]. General flexibility and particularity in addressing voices directly, handling technological issues, and adapting to individual needs were also required. Therapists’ motivation and effort supported feasibility (theme 2), but they stressed that participants needed to feel safe and ready to change, often requiring stability in other life areas. Addressing voice-related trauma was sometimes difficult within the current framework, highlighting the need for trauma-focused approaches [133,136] and the potential of the developmentally focused AV-EXT to more fully address trauma and broader therapeutic goals [30,32].

#### Participant Barriers

For some trial participants, therapy engagement and feasibility were impacted by difficulties with (voice) presence (themes 3‐4), potentially compromising the outcome [34]. Likewise, emotional disconnection was reported by 2 AT participants who discontinued before the avatar dialogues, as the avatar did not match the voice adequately [67]. Furthermore, anxiety and (fear of) voice reactions could disrupt sessions and therapy progression, mirroring therapists’ accounts of situations where the participant or voice(s) would not engage with the intervention in TwV [95]. To mitigate this, a sensitive, individualized approach is essential [29], with tailored exposure and gradual progression to avoid overwhelming responses [40].

#### Organizational Barriers

Limited organizational resources and external workload constrained therapists’ time for training, preparation, and customization (theme 3). Therapists’ extra effort (theme 2) may not be sustainable in routine care, underscoring the need for implementation strategies that address financial support and structural adjustments [137]. The 7-session VRT format is notably shorter than the 16-session minimum for CBTp recommended by guidelines [12], leaving little buffer for the frequent technological malfunctions that disrupted delivery (theme 3). While the usability of a similar digital psychotherapeutic tool may be rated highly under controlled conditions [138], the risk of malfunctions should be considered when evaluating such technologies for real-world use (theme 3). In addition, some trial participants were unable to rehearse avatar recordings at home due to lack of digital devices, highlighting digital exclusion in this population [139]. As there are potential benefits of digital interventions for self-management of symptoms [140], this digital exclusion highlights the research-to-practice gap in digital mental health [53,54].

### Strengths and Limitations

The study’s strengths include its comprehensive exploration of both VRT trial participants’ and therapists’ experiences. Contextualizing the findings within implementation outcomes enhances their relevance for future research, clinical trials, and real-world applications.

Several limitations should be noted. No interviews were conducted with dropouts, limiting insights into reasons for disengagement or discontinuation. However, it is reasonable to assume parallels with AT dropout accounts [67]. Relatedly, those who consented to participate might have had more positive experiences. Furthermore, trial participants’ voices were not interviewed, though this might have offered valuable insights, as explored in TwV [27].

Involvement of people with lived experience was limited to designing the topic guide and validating themes. Greater involvement throughout the research process, including data collection and analysis, might have added important perspectives, challenged implicit assumptions, and enriched interpretations [141]. Co-produced qualitative research on AT [67] and digital interventions [65,66] or more integrated involvement in clinical trials [64] has shown promising potential.

The time between treatment cessation and interviews may have introduced recall bias and memory deficits, potentially leading to biased or inaccurate reporting. However, the sampling strategy prevented the selection of only the most recent completers.

### Future Directions

Advancing VRT requires careful planning for sustainable, potentially commercial delivery models. Ensuring the operational reliability of hardware and software, therapist usability, and user tolerability will be essential, alongside regulatory compliance for integration into routine care. Dedicated implementation studies are needed.

Key VRT challenges include calibrating exposure to balance long-term benefits with the risk of flooding or dropout, with real-time physiological feedback being explored in a pilot trial led by author LBG. Optimizing (voice) presence, or developing strategies to manage suboptimal (voice) presence, remains central for balancing therapeutic impact and safety. Interviewing discontinuers could inform factors influencing engagement, as explored in AT [67].

Careful investment in therapist training, supervision, and standardized materials seems critical for sustaining fidelity and confidence in VRT delivery. However, as the level of experience held by therapists in the Challenge RCT may not be available in routine practice, it is important to examine whether VRT can be effectively delivered by less specialized clinicians, given appropriate training and supervision.

Future research should clarify how VRT compares to other therapies, including its effects on alliance-building and the facilitation of externalization. VRT’s potential for adolescents should be investigated among voice-hearers, with a study currently underway by author DLV. Also, research should clarify what constitutes meaningful outcomes of VRT and establish greater sensitivity in outcome measurement. A forthcoming review will map methodologies for assessing voice content [142].

Investigating therapy experiences across diverse, underrepresented groups is essential. The AVATAR2 qualitative research program includes studies on therapy dropouts and Black individuals’ experiences of AT [143].

### Conclusion

This qualitative study provides comprehensive insights into trial participants’ and therapists’ experience of VRT in the Challenge trial. Several positive aspects of VRT were highlighted, including its validating approach to the voice(s), facilitation of engagement with the voice(s), and the opportunity to share the otherwise private experience of voice-hearing. Embodying the voice(s) in immersive VR-supported avatar role-plays was seen as a key affordance. The collaborative therapist-participant relationship was highly acceptable. Reported positive outcomes included improved voice dialogue, enhanced control, and increased well-being, alongside nuanced changes and experiences not easily captured by quantitative measurements.

VRT was generally perceived as acceptable, appropriate, and feasible. However, several challenges were identified. Content-related issues included instances of suboptimal sense of (voice) presence, limitations to avatar design, and difficulties with reproducing negative voice content. Comfort was affected by technological malfunctions, participant anxiety, exhaustion, and adverse voice reactions. Complexity arose from the demanding nature of the therapy and the nontraditional skills required of therapists. Usefulness could be concealed by measurement insensitivity. Relevance was challenged by tensions between assertiveness and compassion-focused approaches. Furthermore, feasibility seemed constrained by the need for therapists to exert “extra effort” (eg, troubleshooting technology and working beyond scheduled hours), which may be unsustainable in routine care without substantial organizational support (eg, protected time and dedicated technical assistance). Future implementation will require adequate training, supervision, and organizational support.

In conclusion, trial participants’ and therapists’ experiences support VRT as a promising - but challenging - intervention for distressing voices.

## Supplementary material

10.2196/77920 Multimedia Appendix 1 Interview guide for trial participants.

10.2196/77920 Multimedia Appendix 2 Interview guide for therapists.

10.2196/77920 Multimedia Appendix 3 Theme validation workshop.

## Abbreviations

AT: Avatar therapy
AV-BRF: Avatar brief version
AV-EXT: Avatar extended version
RCT: randomized controlled trial
RT: relating therapy
TwV: talking with voices
VR: virtual reality
VRT: virtual reality-assisted therapy
