# Supplementary material for: Immersive Virtual Reality–Assisted Therapy for Distressing Voices in Psychosis: Qualitative Study of Participants’ and Therapists’ Experiences in the Challenge Trial
Source: JMIR Serious Games. 2025 Dec 1;13:e77920. doi: 10.2196/77920 (PMC12670062; doi:10.2196/77920)
Supplement: Multimedia Appendix 1 [file games-v13-e77920-s001.pdf]

Supplementary file A. Interview guide, participants.

Theme	Research question	Interview question	Examples of follow-up questions
Introduction		• How many voices do you hear? How often?<br>• Do they have names?<br>• Which one did you choose to create an avatar of?	*Questions supplemented by data from clinical assessment from the Challenge trial*
The framework for the treatment	Does the participant assess that the conditions for the therapy were acceptable?<br><br>How did the participant perceive the symptom-specific approach?	• There are some set structures for the treatment, e.g., the length of therapy and focus. How were these structures for you?<br><br>• How was it to work with a specific focus on voice hearing?	• How was the length of therapy?<br>• Was 7 sessions sufficient?<br>• Was 1 session/60 minutes a week acceptable?<br><br>• Is it different from other conversations you've had in psychiatry? How?
Motivation for participation	What motivated the participant to take part in the trial?	• What made you want to participate in the trial?	• What expectations did you have before starting?<br>• How was the timing for you life situation?<br>• How did your voice(s) react to you wanting to participate?
Assessment	How did the participant experience the three trial assessments?	• How were the research interviews that you attended before and after the treatment?	• Did you get anything out of them?<br>• Did you find any of the questions too personal?<br>• Was there a difference between participating the first and second time?
VR equipment	How does the participant assess the use of the technical aspects of the VR equipment, including the use of hardware and software?<br><br>What role did the equipment play in the collaboration with the therapist?	• What was your experience with using the VR headset and the computer program?<br><br>• Did you experience any technical issues?<br><br>• Did the equipment have any impact on your collaboration with the therapist?	• How was it to wear the equipment?<br>• How did it affect you?<br><br>• Could you hear the therapist's voice in the background when the avatar spoke? What significance did that have?<br><br>• How was it not being able to see the therapist when you wore the equipment?<br>• Is there anything that could be improved?

VR environment	Was it acceptable to receive treatment in a virtual environment?<br><br>Did the participant experience the VR environment as a reflection of their own private reality?<br><br>What significance does "immersion" have to the participant?	• How was it for you to be inside the virtual world?<br><br>• Was the VR environment different from "the real world"?<br><br>• Were you absorbed by the VR environment?	• Was the (office, beach) environment like reality?<br>• What significance did the (un-) reality have to you?<br>• Was it easy to immerse yourself in?<br><br>• How was the atmosphere? Was it safe or unsafe?<br>• Was it like "another world"?<br><br>• In what way?<br>• Did you forget about time and place?
Avatar design	How did the participant experience the process of designing an avatar?<br><br>Did avatar design active emotions?<br><br>What significance does the embodiment of the voice have?<br><br>Does the visualization encourage certain behaviors or actions?<br><br>How did the participant experience "being seen"/observed by the avatar"?<br><br>How did the participant experience the voice's reaction to the avatar	• Can you describe what it was like for you to create an avatar?<br><br>• Did the design affect how you were feeling?<br><br>• How was it to give the voice a face and a body?<br><br>• How did it affect you to see the avatar in front of you?<br><br>• How did it affect you to "be seen" by the avatar?<br><br>• How did the voice react when you created the avatar?	• Did you think about how the voice would sound before the session? Did it sound/look exactly like the voice? % likeness? What significance did (lack of) personification have? Did it feel real? Which voice did you decide to work with? How was it to only be able to choose one voice?<br><br>• Were you affected by creating the avatar (e.g., anxiety, shame, sadness, anger)?<br><br>• Did it become more concrete?<br><br>• Were you encouraged to do something specific?<br>• Was it overwhelming or confrontational? Did you miss having other senses involved besides sight?<br><br>• Have you experienced this before?<br><br>• Has it reacted this way before? Were you scared?<br>• Did you feel in control?

Avatar dialogue	How did the participant experience the dialogue with the avatar?	• How was it for you to have a conversation with the avatar?	• Can you describe the first meeting? How was the atmosphere? What did you talk about? What did it say? What did you respond?
	Did avatar dialogues active emotions?	• Did it affect how you felt?	• Did you feel sad or scared? Was it dangerous?<br>• Was it different talking to the avatar than the voice? How?
	What significance does one's empathy/imagination have for the interaction? Did the participant accept the premise of the dialogue (as role-playing)?	• Could you immerse yourself in the dialogue with the avatar in VR?	• Did you at any point have to use your imagination? How?
	Did the participant have a sense of agency or mastery?	• Could you (actively) do or say something to the avatar that you couldn't do or say to the voice outside of VR?	• How was it when the dialogue was planned in terms of lines? And when it wasn't?<br>• What did you say? Have you said that to the voice before?
	Did the participant experience the dialogue with the avatar as similar to a game (gamification)?	• Was it more fun or exciting to talk with the avatar inside VR compared to talking with the voice?	• Was it like a game or play?<br>• Was it more motivating?
	What expectations had the participant before avatar dialogues? Was there an expectation build-up before the exposure? Did they get "a taste for more"?	• Did you have any thoughts about what was going to happen before your meetings with the avatar?	• Which? Did they change throughout the sessions?
	How did the participant experience the different phases of the process?	Did the conversation with the avatar change during the process?	• When? How did it change? It what way?

The therapist's role	How was it for the participant to share experiences of voice hearing with the therapist?<br><br>How was it for the participant to share their experiences from VR with the therapist?<br><br>How did the participant experience the therapist's dual role as both supporter and challenger?<br><br>Does the therapy promote a collaboration that is different from other face-to-face therapy?	• How was it to talk about voice hearing and share your experiences with the therapist?<br><br>• How was it to share your experiences from VR with the therapist?<br><br>• How was it for you that the therapist spoke both as themselves and as the avatar?<br><br>• How was the collaboration with the therapist?	• Is it different from other conversations you've had in psychiatry? How?<br><br>• How would it be if there had been no therapist? (Could you do without the therapist, only having the avatar?).<br><br>• How did the therapist help you?<br>• How did the therapist challenge you?<br><br>• Is it different from other conversations you've had in psychiatry? How?
Between therapy sessions	• What impact did the therapy between sessions have?<br><br>• Were participants willing to share their experiences with others?<br><br>• (How) Did the participant use the image of the avatar and the audio recordings?	• Did the treatment affect your daily life between therapy sessions?<br><br>• Have you shared your experiences from therapy with anyone?<br><br>• Did you use the image of the avatar?<br>• Did you use the audio recordings?	• How?<br><br>• Who? What did you share? How was the reaction? Were you met with understanding?<br><br>• How? Did you share with anyone? Reactions? Do you still use it?<br>• Could you hear a difference between the first and last recordings?<br>• Did you share with anyone? Reactions? Do you still use it?

Effect of the therapy, Mechanisms	What does the participant describe as the mechanism of change in their process (if any)?<br><br>Did the treatment facilitate a strong therapeutic alliance?<br><br>Can talking about one's process or voice hearing with loved ones facilitate change?<br><br>Can a changed relationship with the voice (or what the voice says) facilitate change<br><br>Disengagement: Can participants more easily separate what the voice says from what they believe about themselves?<br><br>Has the participant gained insight into the connection between self-perception and voice hearing? Recognition of illness?<br><br>Has the participant experienced mastery and agency as a mechanism of change?	• Has the treatment worked for you?<br><br>• What significance did the collaboration with the therapist have?<br><br>• Have you talked about the project with others?<br><br>• How do you relate to the voice and what it says today?<br><br>• Is what the voice says true?<br><br>• Have you learned something about yourself and your relationship with the voice?<br><br>• Is there something you can do now that you couldn't do before with the voices	• If yes: What has helped you?<br><br>• How did it help you?<br><br>• To whom? What and how did you do it?<br><br>• Did the relationship with the voice/voices change?<br><br>• Has your experience changed?<br><br>• Was there something that "fell into place" during the process?<br><br>• What?

Aftermath:<br>Effects/Side Effects	Can the participant transfer what they have learned to daily life (transfer)?<br><br>Were there any long-term effects or side effects after the treatment?<br><br>How did the voices react to the participant's involvement?<br><br>Did the participant experience alignment between their expectations before participation and the actual process?	•Did you learn something that you have been able to use in everyday life?<br><br>•How has it been since the treatment stopped?<br><br>•What have your voice(s) thought about your participation in the project?<br><br>•Did the treatments align with your expectations? See response from the motivation section.	• Have you learned to handle the voice in a new way?<br>• Have you gained any tools/resources? How are you using them now?<br><br>• Does the effect last?<br>• Did it get better/worse during the process or after?<br><br>• How did the voices react?<br><br>• Have new voices appeared?
The voice	How does the participant describe their relationship with the voice? Do they report/ recognized connections to their personal life?	• Can you tell me a little more about the voice you chose to work with?<br><br>• Are there any connections between you/your life and the voice?	• Have there been any changes?<br><br>• Do you recognize the voice from someone in your life?<br>• Is the voice a part of you?<br><br>*Questions supplemented by data from clinical assessment from the Challenge trial*

Trauma	How did the participant's history with stress or trauma affect the treatment process?	• During or after treatment, have you thought about unpleasant events you experienced as a child/adolescent?<br><br>• Has the treatment reminded you of events from your current or past relationships with other people?<br><br>• Did the treatment remind you of memories from past stressful events?	Before treatment, did you have any thoughts on the connection between voices and stress?<br><br>• During treatment, did any thoughts/memories come up?<br>• Did the treatment bring up past experiences or stressors?<br>• How? How did you work with that?<br><br>*In case relevant:*<br>• Do you think there is a connection between past stressors and the voices?<br><br>*If the participant describes memories/experiences of a traumatic nature:*<br>• Was this something that was focused on in therapy? Did you talk about these memories or the connection?<br><br>• Had you thought about this before the treatment?<br><br>• Did you think about this during the process?<br><br>*Questions supplemented by data from clinical assessment from the Challenge trial*
Negative voice content	How did the therapy affect the degree of negative content in the voice?	• Has the voice started speaking differently to you now?	• Before: What did the voice typically say?<br>• During: What did the avatar say? How did the voice react?<br><br>*Questions supplemented by data from clinical assessment from the Challenge trial*

Control	Does the participant indicate that the degree of perceived control over the voice has changed?	• How would you describe your level of control over the voice now?	• Before you started the treatment: Did you have any control over the voice?<br><br>• During: Any change in control regarding the avatar?<br><br>• Does this apply to one voice or multiple voices?<br><br>*Questions supplemented by data from clinical assessment from the Challenge trial*
Power balance	Do participants experiece the pwoer balance with the voice changes?	• How has the power dynamic between you and the voice been? Has it changed during/after threatment?	• Before: Who was the most powerful?<br><br>• During: Any change?<br><br>• Control: voice/self<br><br>• Follow-up: What is the percentage distribution between you and the voice?<br><br>*Questions supplemented by data from clinical assessment from the Challenge trial*
Self-efficacy	Does the participant feel that they have gained more self-confidence/self-esteem after the intervention?	• How do you feel about yourself – how do you see yourself, evaluate yourself now?<br><br>• Has the voice affected these things?<br><br>• How did you gain the confidence/courage to speak with the voice?	• Before: How was it before the treatment?<br><br>• During: Did anything change along the way?<br><br>• To stand up for yourself?<br><br>• To insist on how you want to be spoken to?
