# Supplementary material for: Immersive Virtual Reality–Assisted Therapy for Distressing Voices in Psychosis: Qualitative Study of Participants’ and Therapists’ Experiences in the Challenge Trial
Source: JMIR Serious Games. 2025 Dec 1;13:e77920. doi: 10.2196/77920 (PMC12670062; doi:10.2196/77920)
Supplement: Multimedia Appendix 2 [file games-v13-e77920-s002.pdf]

Supplementary file B. Interview guide, therapists.

Theme	Research question	Interview question	Examples of follow-up questions
Introduction		• How long have you been part of the trial?<br>• How may therapies have you commenced?	
The framework for the treatment	Do the therepists find that the conditions for the therapy were acceptable and feasible?<br><br>How did the therapists perceive the symptom-specific approach?	• There are some set structures for the treatment, e.g., the length of therapy and focus. How were these structures for you?<br><br>• How was it to work with a specific focus on voice hearing?	• How was the length of therapy?<br>• Was 7 sessions sufficient?<br>• Was 1 session/60 minutes a week acceptable?<br><br>• Is it different from other therapies you've conducted?<br>• How?
Motivation for participation	What motivated the therapists to take part in the trial?	• What made you want to participate in the trial?<br>• Has your motivation changed during the trial?	• What expectations did you have before starting?<br>• How?
Assessment	How did the therepists use data from the three trial assessments?	• Did you use data from the trial assessments?	• How?Did you get anything out of them?<br>• Would you have wanted any any extra data to be provided?
VR equipment	How does the therapist assess the use of the technical aspects of the VR equipment, including the use of hardware and software?<br><br>What role did the equipment play in the therapeutic alliance?	• What was your experience with using the VR headset and the computer program?<br><br>• What role dit the equipment play for the collaboration with the participant?<br><br>• Did the equipment work as is should be?	• How was it to use IT equipment in therapy?<br>• How did it affect you?<br><br>• How did it feel to talk to the participant though a head-set?<br><br>• Did you experience any technical issues?

VR environment	Was it acceptable to deliver treatment in a virtual environment?<br><br>Did therapists experience VR to provide access to participants' (private) world?<br><br>Do therapists experience that VRT enables paticipants to show reactions in the moment and should not describe them retrospectively as in traditional therapy ("show it - don't tell")?<br><br>What significance does the therapist ascribe to "immersion"?	• How was it for you that you were physically sitting with the participant, but they were in the virtual world?<br><br>• Did the virtual environment give you access to something in the participant that would otherwise be inaccessible?<br><br>• Did it have any significance that you could see the participants' reactions to the dialogue from VR?<br><br>• Did you experience the participants to be absorbed by the VR environment?	• In what way?<br><br>• Is it different from other therapies?<br><br>• Is it different from other therapies?<br><br>• Did they forget time and place?<br>• What significance did that have?<br>• Was there a difference between participants who became immersed and those who didn't?
Avatar design	How did the therapist experience the process of designing an avatar?<br><br>What significance does the therapist perceive the avatar's realism to have for the process?<br><br>What significance does the embodiment of the voice have for the therapist and the therapy?<br><br>Does the therapis have any suggestions for improvements or complaints about shortcomings?	• Can you describe what it was like for you to create an avatar of participants' voices?<br><br>• How realistic were the avatars?<br><br>• What was it like for you to see the participant's voice with a face and body?<br>• How did it affect the therapy that the voice had been given a body through the avatar?<br><br>• Was there anything you felt was missing in the program?	• What did it contribute to create an avatar together with the participant?<br><br>• Did it sound/look exactly like the voice?<br>• How many percent was it like the voice?<br>• Did you learn anything? Were there any challenges?<br><br>• Did you learn anything?<br><br>• What did the body contribute with?<br>• Did it make things more concrete?<br><br>*E.g. Children, hairstyle, shadows, animals?*

Avatar dialogue	How was it for the therapist to conduct therapy through the avatar?<br><br>What was it like to reproduce (unpleasant) things the participant normally hears?<br><br>How did it work to say (positive) things the participants normally didn't hear?<br><br>How did the therapist experience their dual role as both support and challenger?<br><br>How did the therapist manage the separation between the avatar and the participant's voice-hearing?	• How was it to work with the participant through the avatar?<br><br>• Can you describe what it was like to reproduce the negative voice content?<br><br>• What was it like to have the avatar say something new to the participant?<br><br>• How was it for you to act as both therapist and avatar?<br><br>• Were you able to separate the avatar from the participant's voice?	• How is this different from other forms of therapy?<br><br>• What did you feel? Was it uncomfortable?<br><br>• What did you feel?<br><br>• Were there any challenges?<br><br>• Did you end up calling the voice the avatar, or the avatar the voice?

Avatar dialogue (continued)	Does the AVATAR promote a collaboration that is greater than in other face-to-face therapies?<br><br>How did the therapist handle unexpected questions from the participant to the avatar?<br><br>Did the therapist experience the dialogue as the avatar to be like a game (gamification)?<br><br>What significance did it have to sit in the same room as the participant?<br><br>How did the therapist assess the intervention?	• How was the collaboration with the participant influenced by the avatar?<br><br>• What did you do if the participant said something to the avatar that you weren't prepared for?<br><br>• Was it more fun or more exciting to speak as the avatar than with your own voice?<br><br>• What was it like to be in the same room as the participant when you spoke together through VR?<br><br>• Have you felt happy/satisfied with delivering the therapy?<br>• Were you satisfied with your own effort?<br>• What were the biggest challenges with the dialogue?	• Was it different from traditional therapy?<br><br>• Did you have to use your creativity? Or imagination?<br>• Did you have to offer interpretations?<br><br>• Did it feel like a game or play?<br>• Was it more motivating?
The therapist's role	What was it like for the therapist when the participant shared their experiences with voice-hearing?<br><br>What was it like for the therapist when the participant shared their experiences from VR?<br><br>Does the therapy promote a kind of collaboration that is different from other face-to-face therapies?	• What was it like to hear the participant talk about their voice-hearing?<br><br>• What was it like to hear the participant talk about their experiences from VR?<br><br>• How was the collaboration with the participant?	• Was it different from other therapy?<br><br>• What significance did it have for the therapy?<br><br>• Is it different from other therapies you've worked with in psychiatry? How?
Between therapy sessions	What impact did the therapy between sessions have?<br><br>How did the therapists experience the use of audio recordings and images between sessions?	• Did you feel that the participant was affected by the therapy between sessions?<br><br>• How did it work with providing the participant with audio recordings and images of the avatar?	• How?<br><br>• Was it used? And how?

Effect of the therapy, Mechanisms	What facilitated change?<br><br>What did not facilitate change?<br><br>Did the participant’s active practice and training facilitate visible change?<br><br>Did the participant’s sense of agency/mastery facilitate change?<br><br>Did talking about their journey or voice-hearing with the therapist or family members facilitate change?<br><br>Did the treatment facilitate change through a strong therapeutic alliance?	• Can you describe a (successful) process where you experienced a change in the participant?<br><br>• Can you describe a process where you did not experience any change in the participant?<br><br>• Did it have any significance that the participant was practicing/repeating something in VR right here and now?<br><br>• Did you experience that the participant gained a sense of control over their actions and the consequences of them?<br><br>• What significance did it have for the outcome if the participant was willing to involve others (you as the therapist and family members) in their voice-hearing?<br><br>• Did the treatment help facilitate an alliance with the participant?	• What worked?<br>• Is it general?<br><br>• What didn’t work?<br>• Is it general?<br><br>• Is it different from other conversations you’ve had in psychiatry? How?<br><br>• What significance did this have?<br><br>• What significance does (not) sharing have?<br>Could it create change?<br><br>• What significance did your alliance with the participant have for the outcome?<br>Was it different from other therapies?
Effect of the therapy, Mechanisms (continued)	Did a changed relationship with the voice (or what the voice says) facilitate change?<br><br>Did recognition of the illness facilitate change?<br><br>Did (achieved) insight facilitate change?<br><br>Did disengagement/defusion facilitate change?<br><br>Did activating the participant’s emotions facilitate change?	• Did you experience a change in the participant's relationship with the voice (not the avatar)?<br><br>• What significance did the participant’s recognition of the illness have for the outcome?<br><br>• What significance does insight have for the outcome?<br><br>• Did participants learn to separate what the voice says from what they think about themselves?<br><br>• Did you experience participants who were emotionally affected?	• How?<br><br>• For example: Has the participant gained insight into the connection between self-perception and voice-hearing?<br><br>• Did it have a different outcome compared to those who didn’t experience this?

Aftermath: Effects/Side Effects	What was the therapist's overall experience of change at the conclusion of the process?<br><br>How was it for the therapist to conclude a process?<br><br>How did the therapist experience the participant's voices reacting to the process?<br><br>Has the therapist learned anything or been surprised by something?	Did you experience positive, negative, or no changes in the participant?<br><br>• How was it for you to conclude a process?<br><br>• Did you experience the participant's voice/voices reacting to the process?<br><br>• Is there anything that surprised you during the process?	• Which ones?<br>• Side effects?<br><br>• Was it different from other therapy? How?<br><br>• How?<br><br>• Have you learned anything from delivering this type of therapy?
The voice	How did the therapist experience the participant's relationship with their voice before/during/after the treatment?<br><br>Did the therapist experience the relationship with the voice as stemming from relational patterns in the participant's life?<br><br>Did the therapist experience the voice as resembling someone from the participant's life history?	• How did you experience the participant's relationship with their voice after they have been in treatment?<br><br>• Did you experience any connections between the participant's relationship with the voice and their relational patterns?<br><br>• Could you recognize the voice from someone in the participant's life history?	• How was it before?<br>• How was it during?<br><br>• If yes: Was it like a specific relationship with a particular person?<br><br>• Did the participant also recognize this?<br>• Did it have any significance for the treatment?
Trauma	How did the participant's history with stressors or trauma affect the treatment process?	• Did you experience that the participant's history with stressors or trauma affected the treatment process?	• Before: Did you have any thoughts about the connection between voices and stressors? For example, after reading the CTQ?<br><br>• During: Did the therapy make you think about the participant's past stressors or trauma? How?<br><br>• Was it something you brought into play during the therapy?

Negative voice content	How did the therapist experience the level of negative content in the voice?<br><br>How did the therapy affect the degree of negative voice content, according to therapists?	• How would you describe the level of negative content in the participant's voice after they have been in treatment?<br><br>• What significance does it have to change the negative content of the avatar's speech?	• How was it before the process?<br>• How was it during the process?<br><br>• Do you experience that it changed the negative content in the participant's voice?
Control	Do therapists experience that the participant's level of control over the voice has changed during the process?	• How would you describe the participant's level of control over the voice after the process?	• How was it before therapy?<br>• How was it during the therapy?
Power balance	What is the therapist's experience of the power dynamics between the participant and the voice?	How did you experience the power dynamics between the participant and the voice after treatment?<br><br>Who is in control?	• How was it before therapy?<br>• How was it during the therapy?
Self-efficacy	Do therapists experience that the participant has gained more self-confidence/self-esteem after the intervention?<br><br>Did the therapist experience that the avatar or the voice affected the self-esteem/self-confidence?<br><br>How did therapists motivate their participants to talk to the voice?	• How would you describe the self-esteem/self-confidence of the participants after they have been in treatment?<br><br>• Could the avatar or the voice affect the participant's self-esteem/self-confidence?<br><br>• Did you do anything to encourage the participant to speak to the voice in the same way as to the avatar?	• How was it before therapy?<br>• How was it during the therapy?<br><br>• Were they able to speak up?<br>• Were they able to insist on how they wanted to be spoken to?
