# Supplementary material for: Immersive Virtual Reality–Assisted Therapy for Distressing Voices in Psychosis: Qualitative Study of Participants’ and Therapists’ Experiences in the Challenge Trial
Source: JMIR Serious Games. 2025 Dec 1;13:e77920. doi: 10.2196/77920 (PMC12670062; doi:10.2196/77920)
Supplement: Multimedia Appendix 3 [file games-v13-e77920-s003.pdf]

Appendix C – Theme validation workshop

Purpose and methods

To validate themes and to ensure they reflected participants' lived experiences, all Challenge trial participants randomized to the experimental group from one trial site (n=44) were invited to a workshop to discuss the 5 generated themes: (1) Using technology to meet the voice, (2) A different approach to voice-hearing and treatment, (3) Limitations, obstacles, and adaptations, (4) Changes, mechanisms and prerequisites, and (5) A price to pay.

Invitations were sent by secure digital postbox (e-Boks) or regular mail. The workshop invitation was also extended as part of an event celebrating the completion of the Challenge trial.

Of the 44 participants invited, eight declined via survey response without providing a reason, and two declined verbally, citing work-related or logistical reasons. Thirty-four did not respond. As no one signed up for the original workshop, the first author, who had planned the event, resorted to convenience sampling. This resulted in two workshops with a total of three Challenge trial participants who provided written informed consent. Additionally, the themes were discussed by telephone with one trial participant who could not attend in person; this participant provided oral informed consent. To reduce the risk of redundancy, none of these individuals had been among the 10 previously interviewed trial participants.

All workshop participants received relevant materials in advance, including: a statement outlining the workshop's purpose; details on the design and content, including a time schedule; a draft of the study manuscript; and a summary of each theme in plain language. Data sources generated from the workshops included: completed templates with participants' reflections on the themes, audio recordings of discussions comparing personal experiences with the generated themes, and written notes by the first author.

Workshops 1 and 2 were held in May 2025, lasting 2 hours and 1 hour and 40 minutes, respectively. Inspired by previous work by the third author, both workshops followed the same structure: an introduction to the study and its purpose; an initial presentation of the generated themes; *Round 1*, where each theme was presented in detail and participants filled out a template reflecting on how their experience with Challenge was or was not represented; a break; and *Round 2,* during which participants discussed their notes for clarification and elaboration. Round 2 discussions were audio recorded, with recordings ranging from 36:24 to 57:34 minutes (mean = 46:59). The first author then clarified their understanding of the participants' overall assessment of the themes for validation. After the workshops, the first author compiled participants' notes from the templates into a document and used the audio recordings for clarification and supplementary detail. Moreover, written notes from a telephone call in May 2025 lasting 32:48 minutes with a trial participant who was unable to attend in person were also incorporated. A detailed description of the workshop design is available upon request from the first author.

Results

Reflections on the generated themes from the workshop participants (WP1–WP3) and the participant who contributed via telephone (WP4) are presented in Supplementary Table 1 (below). Square

brackets containing the first author's notes have been added to clarify the meaning of participants' written reflections and to provide contextual information.

**Supplementary Table 1**. Workshop participants' template notes and first author notes.				
Theme no.	Workshop participant #1	Workshop participant #2	Workshop participant #3	Workshop participant #4
#1 Using technology to meet the voice	**TEMPLATE**<br>[It was a] good thing to see the avatar.<br><br>**RECORDING & NOTES**<br>VR has provided a picture of the avatar that can be used for communicating with the voice. It is valuable to use VR to explore; now you can see the voice.<br><br>As you could not use glasses when wearing the headset, the avatar needed to be very close in order not to be blurred. This provoked anxiety.	**TEMPLATE**<br>Good to see and hear the voice "on the distance" [usually hears voice very close to face].<br><br>Difficult to visually design avatar, when voice is only heard and not seen [only experiences auditory verbal hallucination, not visual hallucination].<br><br>[Challenge therapy] is good for relatives [as it enables understanding through sharing audio recordings of avatar].<br><br>**RECORDING & NOTES**<br>Albeit more realistic, it would have been to anxiety-provoking if avatar was very close to face to begin with. Felt safer due to adjustable distance.<br><br>Moving avatar (voice) away from face and locating it in front of the participant made them more equal.	**TEMPLATE**<br>Lack of diversity [in avatar design].<br><br>Realistic enough [when in the right] mood.<br><br>**RECORDING & NOTES**<br>Voice is like a "shadow person". This could not be made 100% realistic in VR.<br><br>The first sentences in each avatar dialogue were hard to take seriously. But over time, "emotional investment" grew - sometimes triggering anxiety symptoms that made it feel more real.<br><br>Voice interruptions could reduce immersion, creating emotional distance<br><br>Realism could have been improved with a more lifelike avatar and a more authentic, less cheerful environment.	**NOTES**<br>The avatar's voice felt "shadowy" and didn't fully match, but this wasn't distracting as the focus was on the dialogue.<br><br>Sharing recordings with friends was "cool" and helped build new understanding.<br><br>The participant was aware the therapist controlled the avatar, which felt a bit silly. It didn't feel like a real encounter, but they were happy to play along.<br><br>Speaking aloud to the avatar was somewhat embarrassing, so they asked the accompanying person to leave the room.

#2 A different approach to voice-hearing and treatment	**TEMPLATE**<br>Good interaction with the therapist in VR.<br><br>**RECORDING & NOTES**<br>Gained better insight into life and improved their interaction with the voice, now better able to set boundaries and sometimes have a two-way conversation.<br><br>They valued the therapist's support in VR, especially stopping sessions when needed and being present to help manage anxiety. Being in the same room provided safety and helped prevent escalation.<br><br>The participant credited the therapist for enabling them to complete the project.<br><br>Appreciated learning how to better manage their experience.<br><br>Avatar dialogue cannot stand alone: Talking with the therapist before and after was essential.	**TEMPLATE**<br>Therapist = safety. Faster [at establishing] a relation but has always been well received by staff in routine care.<br><br>[Therapist's avatar representation was] good enough but not "hard enough" [milder than voice].<br><br>"Pull yourself together!" [one instance when avatar dialogue was spot on or like the voice - provoking anxiety but also convinced participant that the therapy had potential].<br><br>**RECORDING & NOTES**<br>Therapist provided a sense of safety, acting as a lifeline by being present in the same room (support and trust). Reliance on the therapist as the only lifeline, forced the process of trusting.<br><br>Recordings were especially helpful for relatives who lacked understanding of schizophrenia.	**TEMPLATE**<br>On the same team [participant and therapist working together].<br><br>One is not the only one who has head one's voice [the therapist experiences the voice through the avatar dialogue; relatives through the recordings].<br><br>Having shared the feeling of what the voice says [can in itself be comforting and can create understanding].<br><br>**RECORDING & NOTES**<br>Therapeutic relation is less authoritarian (or hierarchical) as you work in collaboration.<br><br>Important to have the therapist create authentic avatar dialogues – but fully understands that this is nearly impossible.	**NOTES**<br>The participant appreciated the therapist's effort to create an authentic avatar, despite the imperfect voice match.<br><br>They were satisfied with the therapy but uncertain if the benefits came from the collaboration itself.<br><br>VRT helped them confront the voices, unlike medication, but its effects faded after about six months, similar to stopping medication.

		Avatar dialogue cannot stand alone: Talking with the therapist before and after was essential.		
#3 Limitations, obstacles, and adaptations	**TEMPLATE**<br>The voice [avatar's sound] was a bit mechanical.<br><br>Good to have time to make something [the avatar] that looks like [the voice].<br><br>**RECORDING & NOTES**<br>Time was tight and technical issues further reduced this time.	**TEMPLATE**<br>Technology worked well with only one minor instance malfunctioning [no sound in headset – was fixed]<br><br>Design of avatar is more of a feeling, when only hearing [and not seeing] the voice.<br><br>Overall therapy course and individual sessions were too short [flexibility in length requested].<br><br>**RECORDING & NOTES**<br>Too little time in both the number and length of sessions. Flexibility requested to accommodate patient needs, e.g. sessions ranging from 10 minutes to 1.5 hours according to patient's current needs.	**TEMPLATE**<br>Lack of diversity [in avatar design].<br><br>**RECORDING & NOTES**<br>Was rather unaffected by technology malfunctions. Understands that technology sometimes just does not work.	**NOTES**<br>The PC was slow, but this didn't cause much bother.<br><br>The participant recognized that the therapy course was short on time and wished there had been more sessions available.
#4 Changes, mechanisms and prerequisites	**TEMPLATE**<br>Agrees to every point.<br><br>Has gotten a better interaction with my voice.	**TEMPLATE**<br>Make agreements with the voice [unable to do so before therapy – now voice will be quite if asked to, e.g. when	**TEMPLATE**<br>Good, fast results with the voice.<br><br>More predictability [in voice behaviour].	**NOTES**<br>The therapy helped with only one of the three voices - the one represented by the avatar. This voice became

	Has gotten a better insight into the life with the voice.<br><br>**RECORDING & NOTES**<br>Prerequisite: Important to have enough time to complete therapy.	unloading the dishwasher]. This gives more power.<br><br>"Unreliable" when the voice said "I understand that" [Having heard voices since the age of nine, it felt unrealistic that just seven sessions could lead the avatar - or the voice - to respond so differently.]<br><br>Bad period --> Now not forced to do as voice says [participant currently in a bad period, but unlike previous bad periods does not follow orders from voices, e.g. can dismiss the voice's order to study for 18 hours a day].<br><br>**RECORDING & NOTES**<br>Appraises and related to voice content differently now – is taking the voice less serious now.<br><br>Voice communication can also be more dynamic sometime with humour.<br><br>Volume of "own voice" has increased – a	**RECORDING & NOTES**<br>Exponential improvement from session no. 3 and onwards. Started to have two-way communication with voice; it became responsive; more control.<br><br>Learned in what situations voices would appear: Particularly, when tired or lacking energy.	easier to handle, as the participant found it easier to reject what it said. While the content of the voice didn't change, the participant's response to it did.

		positive development.		
#5 A price to pay	**TEMPLATE**<br>A lot of [voice] opposition before, under, and after [therapy sessions].<br><br>A lot of anxiety during initial 4 sessions. Afterwards, it got better. But was very tired afterwards.<br><br>**RECORDING & NOTES**<br>Struggled to attend sessions as the voice tried to block the process and discourage participation, targeting their vulnerabilities. After 4–5 attempts, they felt able to continue, staying with the therapist's support despite thoughts of leaving.<br><br>High anxiety in first 4-5 sessions, but tolerable.<br><br>Extremely tired after first 4-5 sessions – the therapy takes energy.<br><br>Perceived side-effects to be less severe than those stemming from medication.<br><br>More information needed on	**TEMPLATE**<br>Anxiety, especially when creating the avatar [but anxiety not so high as it could have been]<br><br>[Felt] very secure with the therapist.<br><br>Exhaustion – hyperarousal – but that applies to treatment in general [it takes energy to confront avatar/voice].<br><br>[Voice tried to] talk [participant] out [of participating] --> "Cheater" [voice said that participant did not have schizophrenia and would thus ruin the research project by participating].<br><br>**RECORDING & NOTES**<br>Approached the therapy as an opportunity – thus, they tried to push the anxiety away like when doing an exam ('it gets better afterwards').<br><br>Could react with either exhaustion or hyperactivity – with no clear patterns.	**TEMPLATE**<br>Anxiety in each conversation, but reduction towards the conversation's end.<br><br>Too little energy to "hit back" [therapy is draining and makes it harder to respond to voices in the days following].<br><br>Physical reactions [anxiety symptoms when hearing words between-sessions associated with in-session avatar dialogue].<br><br>**RECORDING & NOTES**<br>Expected sessions to be uncomfortable which lead to discomfort before, during and after.<br><br>One instance of intolerable anxiety due to avatar dialogue content going into morbid details – reflecting voice content.<br><br>Voices tried to resist therapy and would want participant to disengage.	**NOTES**<br>*No notes, as participant wanted the call to end at this point.*

	exhaustion as a side-effect before entering therapy.	More information needed on exhaustion as a side-effect before entering therapy.		

Discussion

In the following, the five generated themes are discussed in relation to whether and how workshop participants validated them.

Theme #1 Using technology to meet the voice

WP1 and WP2 agreed with the theme's content on virtual reality (VR) supporting a novel encounter with the voice. For example, WP1 noted the tangibility of having designed the avatar, while WP2 highlighted how being able to place the avatar at varying distances made the interaction easier, as compared to the voice being perceived as very close to the face. In contrast, WP4 was aware of the therapist controlling the avatar and did not think they encountered their voice. Both WP2 and WP3 found that while the realness of the avatar dialogues varied, certain words or phrases could 'hit the spot' and instantly trigger full immersion. While WP2 found it difficult to design the avatar due to being unaccustomed to "seeing" the voice, WP3 found it difficult to design the non-human characteristics of the voice. Both WP2 and WP3 described sharing a recording of the avatar dialogue with a relative as a positive experience - aligning with the theme. No disagreements with the theme content were reported.

Theme #2 A different approach to voice-hearing and treatment

WP1, WP2 and WP3 agreed with the theme content, stating they had good interactions with the therapist both in and outside of VR and felt safe. Aligning with the theme, WP3 found that the traditional hierarchy imbalance between client and therapist was less pronounced, as they were working collaboratively "against" the avatar. All WPs emphasized that the avatar representation was essential for authentic avatar dialogues, stressing the importance of therapists reproducing verbatim voice content rather than milder versions WP4 found it challenging to achieve a matching avatar but noted that they focused on the dialogue itself. No disagreements with the theme content were disclosed.

Theme #3 Limitations, obstacles, and adaptations

WP1, WP2 and WP3 agreed with the theme content, noting that the avatar design had some limitations, such as the avatar not fully matching the voice in sound (WP1) and ambience or appearance (WP3). WP1 emphasized the need for sufficient time during the avatar design phase, while WP2 and WP4 echoed this by suggesting that the duration and number of sessions should be extended due to time constraints – consistent with the theme's point about therapists' experience of time limitations. In line with theme content, all WPs reported only minor technical malfunctions and were relatively unaffected by them. WP1 highlighted a limitation in the headset not being compatible with using glasses, which resulted in the avatar being blurred. No disagreements with the theme content were disclosed.

Theme # 4 Changes, mechanisms and prerequisites

In line with the theme, WP3 found therapy facilitated positive changes quickly. Aligning with another theme aspect, WP1 and WP2 agreed that outcomes varied and could be subtle and difficult to articulate. In line with the theme, WP1 reported improvements in their interaction with the voice, specifically being able to have dialogues and make agreements with the voice, thus, gaining a degree of control. WP3 gained a new understanding of the voice and found it to be more predictable. WP4 noted improved ability to manage the specific voice they chose to represent with an avatar, though this did not extend to other voices. WP4 also observed that the positive effects tended to diminish over time. No disagreements with the theme content were disclosed.

Theme #5 A price to pay

WP1, WP2, and WP3 agreed with the theme content, reporting experiences of anxiety, particularly during the initial sessions. To WP3, anxiety would extend beyond therapy sessions when reminded of specific content from the avatar dialogues. WP1 felt tired after therapy, and WP3 had less energy to withstand the voice after sessions. WP2 reflected that such strong emotional reactions were not unfamiliar and aligned with previous treatment efforts involving the voice. All WPs described that one or more voices had tried to dissuade them from participating in the trial and attending sessions. No disagreements with the theme content were disclosed.

Strengths and limitations

A key strength of the workshop was the inclusion of Challenge trial participants who were not among the original 10 trial participants interviewed. This participant involvement enhanced the validity of the results. However, there were limitations. Due to pragmatic constraints, only participants from one trial site were invited to take part. Initially, none responded, and ultimately only a few participated in the workshop, which may limit the reliability of the validation process.

Conclusion

In conclusion, the generated themes were validated and nuanced through the personal reflections of workshop participants.
